# Small Protein Domains as Potential Spin Labels for In Vitro, Cellular, and Light-Induced Dipolar EPR Spectroscopy

**DOI:** 10.1021/jacs.5c01875

**Published:** 2025-06-23

**Authors:** Andreas Günter, Susanna Ciuti, Lukas Denkhaus, Anna Sappler, Laura Orian, Stefan Gerhardt, Oliver Einsle, Stefan Weber, Marilena Di Valentin, Erik Schleicher

**Affiliations:** † Institut für Physikalische Chemie, 9174 Albert-Ludwigs-Universität Freiburg, Albertstr. 21, 79104 Freiburg, Germany; ‡ Dipartimento di Scienze Chimiche, Università degli Studi di Padova, via Marzolo 1, 35131 Padova, Italy; § Institut für Biochemie, Albert-Ludwigs-Universität Freiburg, Albertstr. 21, 79104 Freiburg, Germany

## Abstract

This study explores the potential of small light-oxygen-voltage (LOV) domains for utilization as protein spin labels in different dipolar EPR spectroscopy methods. The distinctive photochemical properties of selected LOV domain variants are exploited to generate a variety of (meta)­stable flavin mononucleotide (FMN) radicals upon blue light absorption. Three different radicals, FMN^·–^, FMNH^·^, and an FMN-methionine radical, and an excited FMN triplet species, were generated. The FMN radicals were generated in LOV single domains and two model LOV1-LOV2 fusion proteins, and the latter proteins demonstrated that simple and effective orthogonal spin labeling can be performed. Subsequently, dipolar EPR experiments were conducted in aqueous solution and in cells with and without additional light excitation, in order to measure the distances between the FMN cofactor radicals, and to infer the structure and dynamics of the LOV domain proteins. Interestingly, all LOV1-LOV2 fusion proteins exhibit defined but largely distinct distances. This can be attributed to two factors: the respective LOV domains have different interactions with each other, and the presence of neutral FMN radicals leads to dimerization of the LOV1 domains. Nevertheless, using LOV domains as genetically encoded spin labels could offer numerous advantages. As a true molecular biology concept, labeling and measurements can be performed in any accessible cell type using light as the only stimulus. Additionally, the various paramagnetic FMN states enable the measurement of distances between two radicals, as well as between a radical and a triplet state.

## Graphical abstract

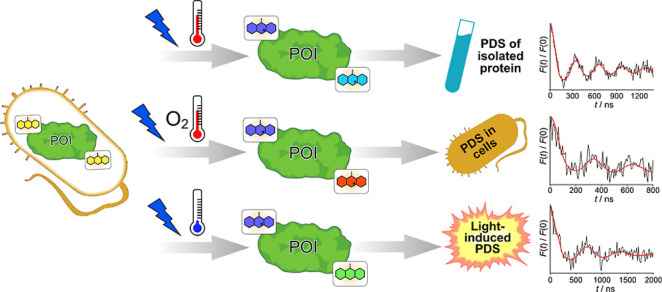

## Introduction

Pulsed dipolar spectroscopy (PDS) has become a major application in the field of electron paramagnetic resonance (EPR) spectroscopy of biomolecules.^1^ Using a variety of pulse sequences, it can be employed for straightforward quantitative distance determination, including the analysis of distance distributions, provided that effective methods for selective spin labeling are available.^2^ In addition to the already established pulsed electron–electron double resonance technique (PELDOR, also termed double electron–electron resonance or DEER),^3^ recent publications have described and introduced light-induced PDS for determining the dipolar coupling between short-lived excited triplet states and stable radicals.^4^

The utilization of photogenerated triplet states as spin labels may offer a number of potential advantages over the use of stable radicals. The generation of short-lived triplet states by photoexcitation of chromophores with a diamagnetic ground state renders them switchable, and numerous protein families have cofactors for which the triplet state could be used as an endogenous probe. Moreover, the population of an excited triplet state originates from intersystem crossing and thus does not follow the Boltzmann distribution. Consequently, photoexcited triplet states are strongly polarized, which can lead to a significant enhancement in sensitivity when coupled with certain light-induced PDS techniques.^4d^ To date, there are only a few pulse sequences capable of measuring the distance between a triplet state and a stable radical. One of these is the light-induced PELDOR sequence, in which a light pulse for triplet generation precedes the PELDOR sequence and the triplet signal is observed.^4d^ The disadvantage of this sequence is that there is a large orientation selection and only a small fraction of the light-excited molecules contribute to the PELDOR signal. However, this disadvantage may be compensated by the large electron spin polarization of the triplet state. Another technique called laser-induced magnetic dipole spectroscopy (LaserIMD) has been introduced.^4b^ In LaserIMD, the dipole–dipole coupling is introduced by triplet excitation at variable times during the observer pulse sequence. The experiment is conducted by applying a Hahn echo sequence to the radical, with the objective of recording the primary echo while changing the position of the complete pulse sequence in relation to the fixed position of a laser flash. When the laser flash occurs at a time *t* before the refocusing of the radical echo, the dipolar interaction between the two spins creates a phase offset at the time of echo formation. This results in a modulation of the radical echo as a cosine function of the dipolar frequency. The technique has been improved in terms of zero-time determination by introducing an additional π pulse and detecting the refocused echo of the stable radical (refocused LaserIMD, ReLaserIMD).^4e^ Nowadays, metal-containing tetrapyrroles and carotenoids,^4b,4d,5^ covalently attached organic dyes,^6^ and bound fullerenes^7^ have successfully been used as photoswitchable triplet spin labels for distance determinations in proteins.

Although PDS now provides a number of options for determining distances and their distributions in biomolecules, a straightforward application for in-cell distance measurements is essential to compete with other dipolar spectroscopy techniques, such as FRET, in particular in combination with microscopy techniques.^8^ The PELDOR experiments of proteins in biological cells present a significant challenge. The difficulty lies in the introduction of an adequate concentration of labeled protein into the cell, or alternatively in the labeling of proteins directly inside the cells. The negative redox potential of the cytosol prevents the use of conventional nitroxide spin labels.^9^ To date, the application of PELDOR spectroscopy in cells has been achieved in a limited number of studies recently reviewed by Pierro and co-workers,^10^ using highly specialized techniques.

The first approach is to attach spin labels to the isolated and purified protein of interest (POI) which is then delivered to a certain cell type. The microinjection of labeled proteins directly into cells has been shown to be an effective method for PELDOR experiments, although this approach is currently limited to a few large cell types, such as oocytes.^11^ Other techniques for delivering spin labeled proteins to different cell types cells are electroporation,^12^ heat shock,^13^ and osmotic shock.^14^ The obvious drawback of all these techniques is that the POI has to be first isolated, purified, spin labeled and incorporated into the cells. In particular, the heat shock method may be problematic for temperature-sensitive proteins. A different approach is the delivery of copper–nitrilotriacetic acid (Cu­(II)–NTA) complexes.^15^ The POI binds two Cu­(II)–NTA complexes via two specifically placed Histidine motifs (dHis) and the distance between the copper ions can then be measured by PELDOR spectroscopy. Membrane proteins with exposed Cysteines may be accessible for on-cell spin labeling, however, this method is limited to the direct lipid environment of membrane proteins.^9b^ A more sophisticated method is the usage of unnatural amino acids which often possess an azide, alkyne or tetrazine function in their side chains.^16^ The spin label is then a small molecule which is delivered to the cell and is attached to the unnatural amino acid by click chemistry or cycloaddition reactions. These promising approaches allow the spin labeling reaction directly inside the cells, followed by successful in-cell PELDOR experiments.^16c,17^

In this study, we introduce a novel concept of utilizing small cofactor-dependent proteins as spin labels. This concept is particularly well-suited to addressing the current limitations of in-cell EPR spectroscopy. The fusion of protein-based spin labels with the POI at the DNA level enables the expression and spin labeling to occur within any cell which is suitable for protein expression. The so-called light-oxygen-voltage (LOV) domains have been selected as proof-of-principle protein spin labels. LOV proteins represent a class of sensory blue light photoreceptors that mediate diverse physiological responses in archaea, bacteria, protists, fungi, and plants.^18^ The response is carried out by the relatively small FMN-binding LOV domain, which belongs to the PAS superfamily.^19^ In phototropins, two LOV domains are linked to a C-terminal kinase domain whose activity is controlled by photochemical processes.^18a^ The reason why two LOV domains are required for their control is not yet fully understood. In general, the structures and photochemical processes of LOV domains are highly conserved.^18a,18b^ In addition to their prominent role in nature, LOV receptors also serve as genetically encoded actuators in optogenetics, enabling the spatiotemporal precise control of cell states and processes by light.^20^

In LOV domains, following the absorption of light by the initial oxidized FMN_ox_ redox state, a covalent bond (the so-called cysteinyl-4a-adduct) is formed between a highly conserved cysteine residue and the C4a atom of the flavin-isoalloxazine ring system, presumably via a triplet-generated radical-pair intermediate.^21^ Upon mutagenesis of the conserved cysteine to alanine, this blue light-induced adduct formation is no longer possible.^21b^ However, the LOV domain retains its photoreactivity (Figure 1A) and a short-lived triplet state (^3^FMN) intermediate is instead populated upon blue light excitation due to efficient intersystem crossing.^21b^ This is followed by electron transfer between the FMN and a Trp or Tyr residue, resulting in the formation of an intermediate radical pair.^22^ The addition of an electron donor effectively reduces the amino acid radical, leaving a FMN radical, which is present in its protonated form (FMNH^·^) and is long-lived under anoxic conditions. A similar photochemical process occurs at low temperatures, but an FMN anion radical (FMN^·–^) may be formed (see below). Due to these photochemical properties, alanine mutants of LOV domains can be employed either as radical or triplet spin labels. Replacing the cysteine at position 57 in LOV1 with a methionine residue also has significant impact on the photochemistry of the protein. Following irradiation with blue light a methionyl-flavin adduct is irreversibly formed (Figure 1B).^23^ A reaction mechanism for its formation was proposed on the basis of electron–nuclear double resonance spectroscopy (ENDOR) data, which proceeds via radical intermediates, a rearrangement, and a final oxidation step. A covalent bond is irreversibly formed between the N5 atom of the FMN and the methyl group of the methionine. This neutral radical (FMN^·^-Met) species exhibits a significant red shift of the absorption bands compared to neutral FMN radicals and is stable even under aerobic conditions. These properties render this variant ideally suited as a permanent radical spin label. Thus, by carefully selecting the reaction conditions, such as the temperature and the presence or absence of oxygen, different FMN radicals may be generated in LOV domains, in each case using light as the only external stimulus.

**Figure 1:**
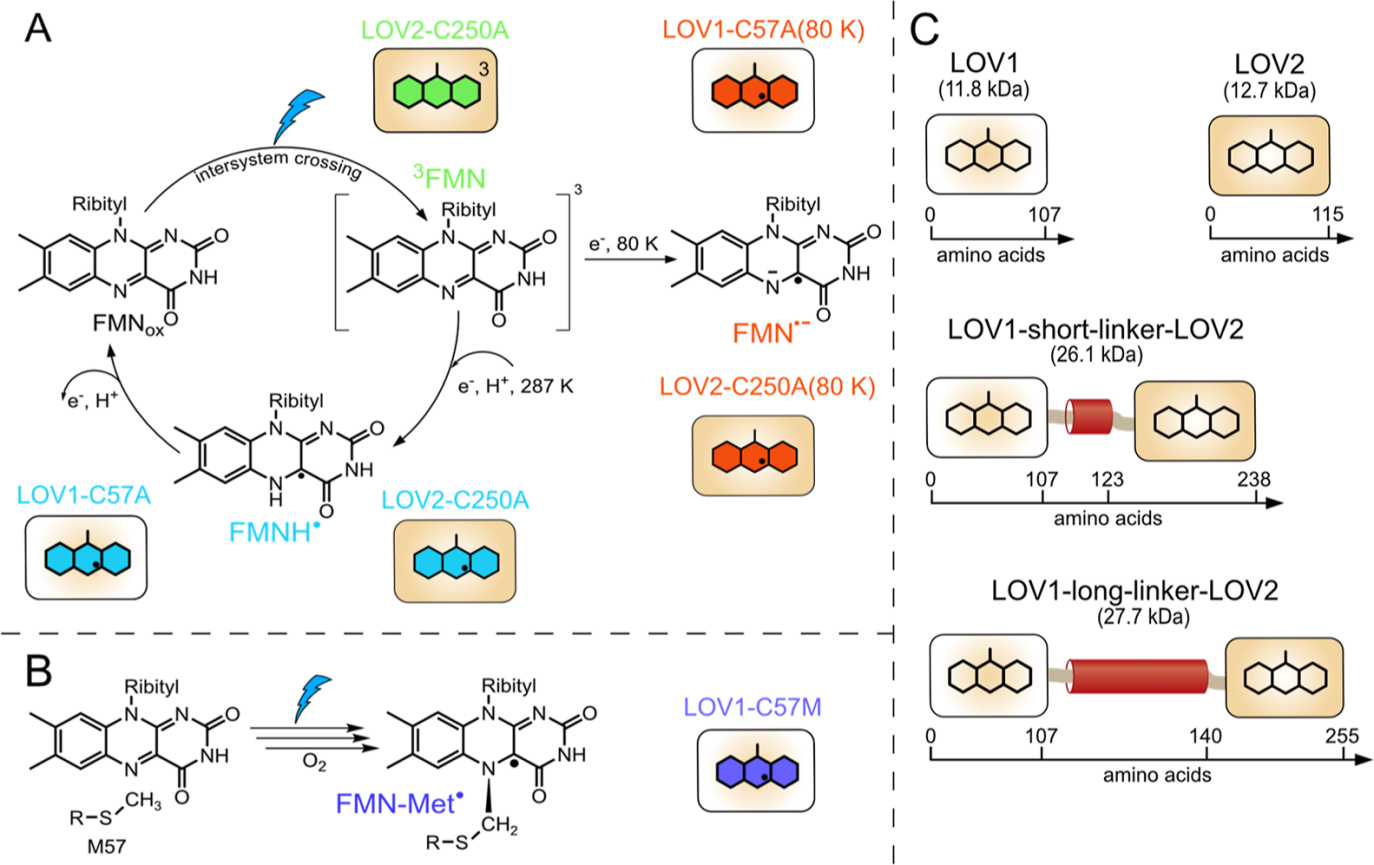
Photochemistry and FMN radical generation in various LOV domain variants (LOV1 domains are shown in pale ochre, LOV2 domains are shown in ochre), including a color scheme of the different FMN radicals. (A) Photochemistry of LOV1 and LOV2 C → A variants: following blue light excitation, a short-lived ^3^FMN intermediate (green) is generated, which is subsequently reduced. At low temperatures, no proton transfer occurs and FMN^·–^ (red) is stabilized, whereas a protonated FMNH^●^ (light blue) is formed at room temperature. Both radicals exhibit prolonged lifetimes under anoxic conditions. (B) Following blue light irradiation of the LOV1 C57M variant under aerobic conditions, a methionyl-flavin adduct radical (dark blue) is irreversibly formed. (C) Schematic illustration of the LOV domains and the LOV-LOV fusion proteins including their respective sequence lengths. The lengths shown are not to scale.

The present study thus aims to assess the suitability of LOV domains as versatile protein-based spin labels. The objective is not to ascertain the fundamental ability of these domains for distance determination; rather, it is to facilitate their broad applicability. Consequently, not only stable radicals but also transient paramagnetic triplet states are generated to test different PDS methods. In this way, we also explore for the first time the potential of an endogenous FMN cofactor to act as a triplet spin label. Measurements are performed not only in solution but also in cells, and in addition to identical radical species, orthogonal labeling strategies are employed.

## Results

### Design and Production of LOV Domains and Model Fusion Proteins

All protein constructs used are derived from the Phot1 protein of , which is a 75 kDa protein consisting of two LOV domains (LOV1 and LOV2) with an approximately 75 amino acid long linker region in between, and a C-terminal serine/threonine kinase. Different single LOV1 and LOV2 domains were produced, and additionally, two LOV1-LOV2 fusion proteins with synthetic α-helical linkers of different lengths were designed and produced (Figure 1C). In all constructs, the conserved cysteine residues essential for cysteinyl-4a-adduct formation were replaced by either alanine (LOV1-C57A and LOV2-C250A) or methionine (LOV1-C57M) residues. Optical spectroscopy and gel filtration chromatography showed that all LOV single domains could be produced as folded proteins with bound FMN cofactor in high yields (typically 0.6–1 mg/g wet cells were obtained from ). Based on the reaction schemes in Figure 1, the respective FMN radicals were prepared.

The model fusion proteins are composed of a LOV1-C57M and a LOV2-C250A domain. The rationale behind this design is that the FMN^·^-Met radical of the LOV1 domain always acts as a stable first radical, while, depending on the reaction conditions, the FMN of the LOV2 domain can be prepared as FMN^·–^, FMNH^·^, or as ^3^FMN, allowing simple and effective different orthogonal labeling procedures. The connection and simultaneous spatial separation of the LOV domains was achieved by using α-helical linkers consisting of a periodic (EAAAK)_*n*_ amino acid motif, which has previously been successfully employed to modulate distances between fluorescent proteins.^24^ Two different linker lengths were produced, one with *n* = 2 (short linker, SL) and the other with *n* = 5 (long linker, LL). After purification, all fusion proteins were obtained in a folded state, each with two FMNs bound, as revealed by optical spectroscopy. Therefore, it can be assumed that defined conformations are present in each case. All proteins and fusion proteins used, their preparation and the abbreviations assigned to each construct are summarized in Figure 1C and in Table 1.

**Table 1:** Sample Preparation, FMN Redox States, and Abbreviations Used

sample^a^	FMN redox state	abbreviation	sample preparation
LOV1-C57M	FMN^·^-Met	1[FMN^·^-Met]	blue light illumination at room temperature, freezing in liquid nitrogen
LOV1-C57A	FMNH^·^	1[FMNH^·^]	blue light illumination at room temperature, freezing in liquid nitrogen
LOV2-C250A		2[FMNH^·^]	
LOV1-C57A	FMN^·–^	1[FMN^·–^]	blue light illumination at 80 K
LOV2-C250A		2[FMN^·–^]	
LOV1-C57M-SL-LOV2-C250A	FMN^·^-Met/FMNH^·^	1[FMN^·^-Met]-2[FMNH^·^]	blue light illumination at room temperature, freezing in liquid nitrogen
LOV1-C57M-LL-LOV2-C250A		1[FMN^·^-Met]···2[FMNH^·^]	
LOV1-C57M-SL-LOV2-C250A	FMN^·^-Met/^3^FMN	1[FMN^·^-Met]-2[^3^FMN]	blue light illumination at room temperature, reoxidation in air, freezing in liquid nitrogen, laser illumination at 80 K
LOV1-C57M-LL-LOV2-C250A		1[FMN^·^-Met]···2[^3^FMN]	
LOV1-C57M-SL-LOV2-C250A	FMN^·^-Met/FMN^·–^	1[FMN^·^-Met]-2[FMN^·–^]	blue light illumination at room temperature, reoxidation in air, freezing in liquid nitrogen, blue light illumination at 80 K
LOV1-C57M-LL-LOV2-C250A		1[FMN^·^-Met]···2[FMN^·–^]	
Samples for Crystallization			
LOV2-C250A	FMN_ox_		sample preparation in the dark
LOV1-C57M-SL-LOV2-C250A	FMN^·^-Met/FMN_ox_	1[FMN^·^-Met]-2[FMN]	blue light illumination at room temperature, reoxidation in air

a SL = short linker/LL = long linker.

### PDS of Single LOV Domains

A series of single LOV domains were prepared in well-defined FMN oxidation states and investigated by PELDOR spectroscopy to analyze their EPR properties and quaternary structures (Figure 2 and Table 1). The following samples were subjected to investigation: the LOV1-C57M domain was examined both immediately following exposure to blue light and after subsequent incubation in the dark; the LOV1-C57A and the LOV2-C250A domains were either illuminated by blue light at room temperature (RT) and frozen directly afterward, or illuminated by blue light at 80 K. Initially, the relaxation behavior of the individual domains with different FMN radicals was investigated to ascertain their suitability as spin labels for PDS. At 80 K, phase memory times in the range of 1–2.5 μs were determined (Figure S1), allowing for time trace of about 3 μs, which can be sufficient to detect distances of up to 5 nm.^25^ All PDS time traces were evaluated using Tikhonov regularization.^26^ Consequently, even in the absence of defined distances within the investigated experimental samples, the analysis invariably yields a distribution of distances. Accordingly, a distance distribution comprising a minimum of three distinct distances is considered to represent a nondefined quaternary or a monomeric structure.

**Figure 2:**
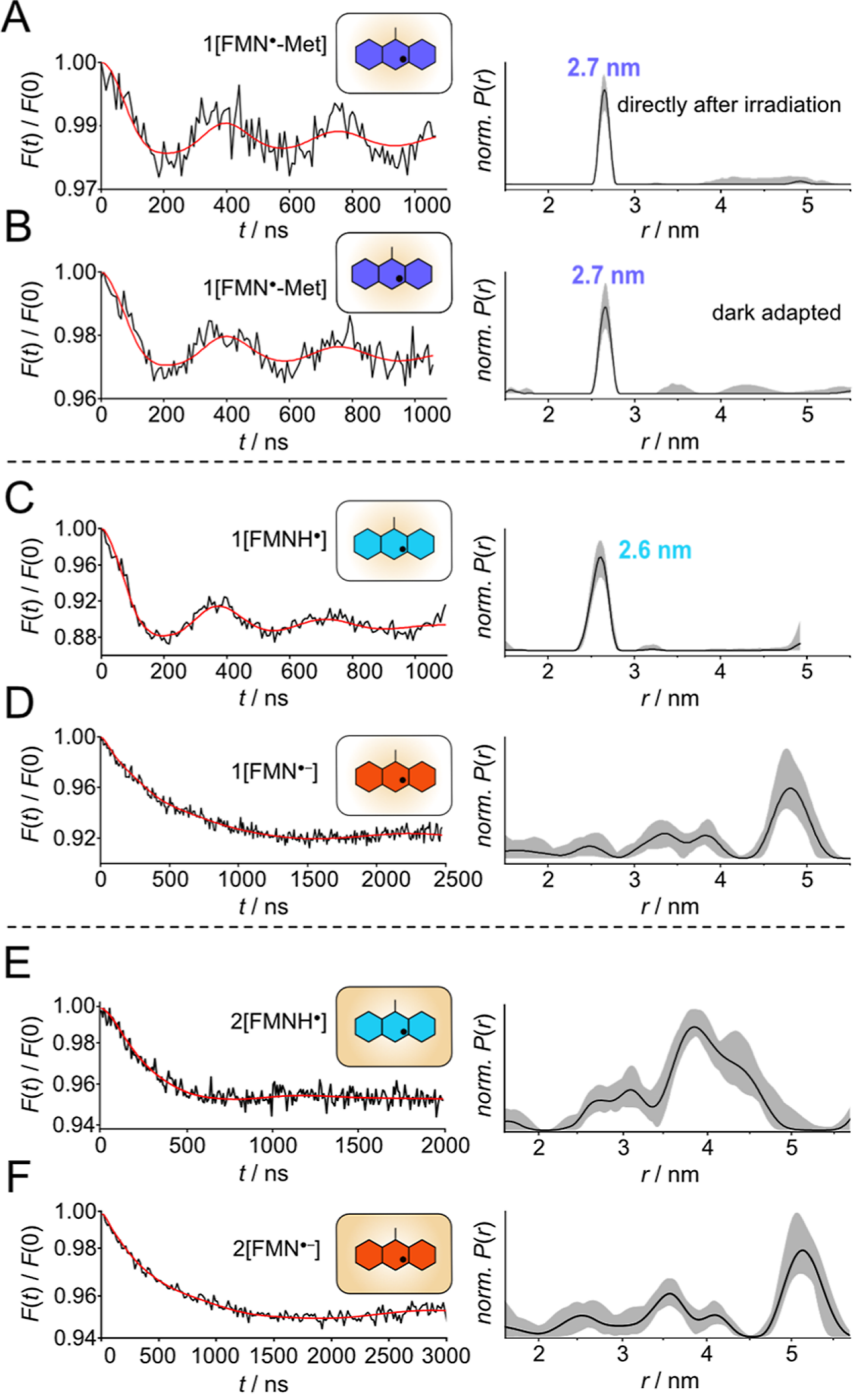
PELDOR spectroscopy of single LOV domains. Background-corrected time traces (black) and corresponding fits (red) of indicated FMN radicals in LOV domains are shown on the left; the corresponding distance distributions, as obtained by Tikhonov regularization with DeerAnalysis, are shown on the right. The gray shaded areas correspond to the error region from the Tikhonov validation. (A) 1­[FMN^·^-Met] directly after illumination, (B) 1­[FMN^·^-Met] after incubation in the dark, (C) 1­[FMNH^·^], (D) 1­[FMN^·–^], (E) 2­[FMNH^·^], and (F) 2­[FMN^·–^].

Analysis of the PELDOR time traces of 1­[FMN^·^-Met] samples frozen directly after illumination or frozen after illumination and incubation in the dark showed an identical distance of 2.7 nm with a narrow distance distribution (Figure 2A,B). As the published structure is monomeric,^27^ the 1N9O data set was subjected to further analysis using the PISA software.^28^ Based on the surface properties of the protein and subsequent thermodynamic calculations, the software is capable of determining the quaternary structure of the biologically active form of a protein. In the case of LOV1-C57M, a dimer is postulated, with a distance of 2.65 nm between the two FMN C4a atoms. This is in excellent agreement with the experimentally determined distance (Figure S2 and Table 1). Both sample preparations showed identical distances with comparable narrow distribution curves, indicating that identical dimers are present.

Given the effective reduction of the FMN in the 1­[FMN^·^-Met] sample and the air-stability of the generated FMN-methionine radical,^23a^ it is not feasible to investigate this variant in its FMN_ox_ redox state. To ascertain whether LOV domains exist as dimers in all paramagnetic oxidation states, the PELDOR experiments were repeated with a LOV1-C57A and a LOV2-C250A sample. Both variants were effectively photoreduced to the semiquinone oxidation state in the presence of an external electron donor and were also fully reoxidized in the presence of oxygen (Figure 1 and Table 1). Two distinct samples were prepared for each of the two variants: one illuminated at RT and subsequently frozen (1­[FMNH^·^] and 2­[FMNH^·^]), and the other illuminated at 80 K (1­[FMN^·–^] and 2­[FMN^·–^]).

The PELDOR experiments for the 1­[FMNH^·^] and 2­[FMNH^·^] samples unexpectedly yielded different distance distributions (Figure 2C,E). Sample 1­[FMNH^·^] resulted in a very similar distance (2.6 nm) to that of the dimeric 1­[FMN^·^-Met] sample (2.7 nm). When analyzing the PELDOR trace of the 2­[FMNH^·^] sample (Figure 2E), a very broad distance distribution between ∼2.5 and 5 nm was observed. This indicates that the sample’s most likely conformation is an undefined quaternary structure without specific interactions. The analysis of the PELDOR time traces of the 1­[FMN^·–^] and 2­[FMN^·–^] samples also revealed a large number of different distances (Figure 2D,F), with large uncertainty after validation, which would be explained by monomeric proteins. Any distance larger than ∼5 nm cannot derive from dimerization, since the size of both LOV domains does not permit such large distances when they are in direct contact (see Figures S2 and S9), and no shorter distances are detected. Consequently, three sample preparations, 1­[FMN^·–^], 2­[FMN^·–^] and 2­[FMNH^·^], are considered monomeric and thus distinct from 1­[FMN^·^-Met] samples.

After preparation, the 2­[FMN^·–^] sample (Figure S3) was additionally analyzed by proton ENDOR spectroscopy, and compared with the results of a 2­[FMNH^·^] sample (Figure S4). An increase in the H8α hyperfine coupling constant by 3.5 MHz and the absence of the large hyperfine coupling of the nitrogen-bonded H5 proton are evident. Both values can be used to differentiate between flavin neutral radicals and anion radicals.^29^ Therefore, there is clear evidence for the presence of an unprotonated FMN^·–^ radical.^30^ The photochemical details of LOV2-C250A were elucidated by low-temperature transient EPR spectroscopy (Figure S5). As is typical for other LOV domains, effective formation of the ^3^FMN occurs within microseconds after light excitation, and spectral simulations of the triplet state signal yield similar parameters to literature values.^21b^ Subsequently, the broad triplet signal decays within a few microseconds and a much narrower signal remains. This spectrum was well described by spectral simulations assuming a relaxed radical pair formed from a triplet precursor, consisting of a FMN and a Tyr residue, separated by approximately 1 nm.^31^ These findings, when combined, indicate that electron transfer, but not proton transfer takes place after light excitation at 80 K.^21b^

### PDS of LOV1-LOV2 Fusion Proteins

The next step was to investigate the LOV1-C57M-LOV2-C250A fusion proteins. The FMN redox state in the LOV2-C250A domain can be flexibly tuned because the FMN of the LOV1-C57M domain is already present in the stable FMN^·^-Met radical following protein production (or short blue light irradiation). The production of 2­[FMNH^·^] can be achieved by freezing the protein directly after blue light irradiation, while FMN_ox_ for the ReLaserIMD experiment can be produced by incubating the protein in the dark for several hours. Finally, FMN^·–^ can be generated by irradiating FMN_ox_ with blue light at 80 K. The respective redox states can be monitored by UV–vis spectroscopy (Figure S6) as the absorption bands at 448, 570, and 675 nm correspond to marker bands of FMN_ox_, FMNH^·^, and FMN^·^-Met, respectively.^23b,32^

Figure 3 illustrates the time traces and respective distance distributions obtained from the PDS experiments conducted on the 1­[FMN^·^-Met]-2­[FMNH^·^], 1­[FMN^·^-Met]-2­[FMN^·–^] and 1­[FMN^·^-Met]-2­[^3^FMN] fusion proteins. A discernible modulation of the dipolar traces was observed for all three combinations of FMN redox states. The modulation depths differ between the FMN radical combinations (about 17.8% for FMNH^·^, 5.4% for FMN^·–^ and below 1% for ^3^FMN), but all values confirm the presence of a substantial radical/triplet concentration of the two FMNs and sufficiently long relaxation times for a reliable analysis. The analysis uncovered a single, narrow peak in the distance distribution in each case. It is noteworthy that different combinations of FMN radicals yielded disparate distances. The investigation of the 1­[FMN^·^-Met]-2­[FMNH^·^] sample resulted in a distance of 2.5 nm (Figure 3A), while the 1­[FMN^·^-Met]-2­[FMN^·–^] sample yielded a distance of 3.4 nm (Figure 3B). From the ReLaserIMD experiment, a distance of 3.2 nm between the FMN^·^-Met and ^3^FMN was obtained (Figure 3C). The observed low modulation depth of this experiment can be partially attributed to the prolonged laser irradiation, which resulted in the photoreduction of FMN_ox_ to FMN^·–^ (see Figure 3 and also Methods section for details of the FMN^·–^ preparation).

**Figure 3:**
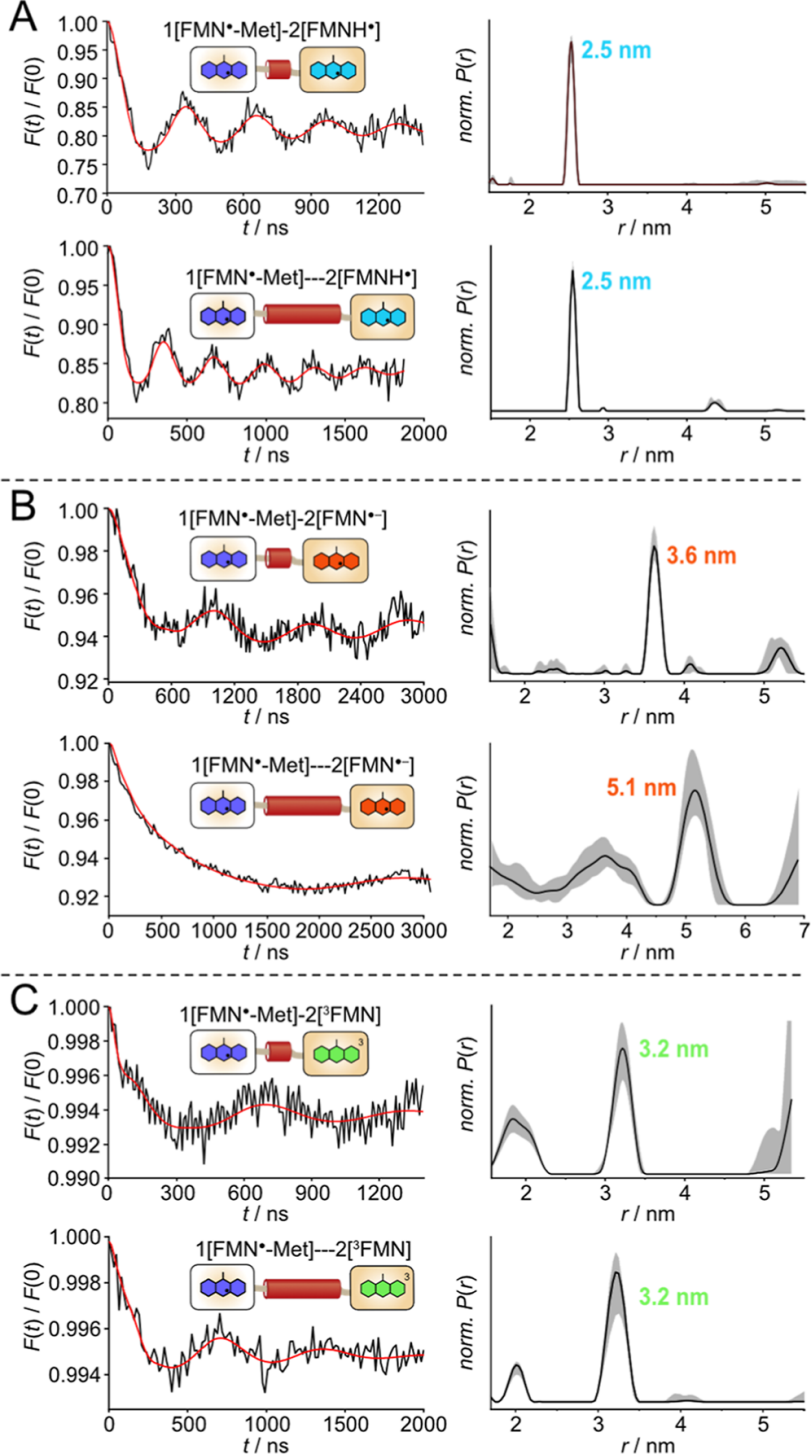
PELDOR and LaserIMD spectroscopy of LOV–LOV fusion proteins with indicated FMN radicals and linker lengths. Background-corrected time traces (black) and corresponding fits (red) of indicated LOV fusion proteins are shown on the left; the corresponding distance distributions, as obtained by Tikhonov regularization with DeerAnalysis, are shown on the right. The gray shaded areas correspond to the error region from the Tikhonov validation. (A) 1­[FMN^·^-Met]-2­[FMNH^·^] and 1­[FMN^·^-Met]···2­[FMNH^·^], (B) 1­[FMN^·^-Met]-2­[FMN^·–^] and 1­[FMN^·^-Met]···2­[FMN^·–^], (C) 1­[FMN^·^-Met]-2­[^3^FMN] and 1­[FMN^·^-Met]···2­[^3^FMN].

The next objective was to examine the 1­[FMN^·^-Met]···2­[FMNH^·^], the 1­[FMN^·^-Met]···2­[FMN^·–^], and the 1­[FMN^·^-Met]···2­[^3^FMN] samples, which contain the long linker. Once more, visible modulations were discernible in all dipolar traces. The ensuing analysis yielded narrow distance distributions, with a single distance observed in each case. In the case of 1­[FMN^·^-Met]···2­[FMNH^·^] and 1­[FMN^·^-Met]···2­[^3^FMN] (ReLaserIMD experiment), the constructs with long linkers exhibited identical distances to the ones with short linkers (Figure 3A,C), indicating that the length of the linker has no influence on the respective FMN···FMN distance. The 1­[FMN^·^-Met]···2­[FMN^·–^] construct resulted in a distance of 5.1 nm, which is 1.4 nm longer than that of the construct with the short linker (Figure 3B). Therefore, it can be concluded that the longer linker exerts an influence on the distance only when LOV2-C250A is in the FMN^·–^ redox state.

### In-Cell PDS of LOV1-LOV2 Fusion Proteins

A 1­[FMN^·^-Met]-2­[FMNH^·^] sample was also employed in an experiment conducted on intact cells. The preparation of the FMN redox states was performed directly after cell cultivation (see Experimental section). In this experiment an additional mutation (W291F, this construct is designated as 1­[FMN^·^-Met]-2­[FMNH^·^]^*^) in the LOV2 domain that allows for a more efficient photoreduction was used (Figure S7). Irradiation was conducted at 4 °C, and the cells were shock-frozen in liquid nitrogen. Under the microscope, the cells showed no difference in terms of shape, mobility or division properties before and after light exposure. Continuous wave (cw) EPR control experiments revealed that unirradiated cells exhibited an EPR signal originating from the stable FMN^·^-Met radical, which increased by about 50% after blue light illumination, due to the formation of the FMNH^·^ radical (Figure S8). The PELDOR experiment was carried out in cells, and as a control, with isolated protein in frozen solution under identical experimental conditions (Figure 4). The PELDOR time traces show detectable modulation, however, the modulation depth (17.4% in solution versus 2.4% in cell, Table 2) and signal-to-noise ratio (32.3 versus 3.8) of the in-cell experiment is lower than the one observed in solution.

**Figure 4:**
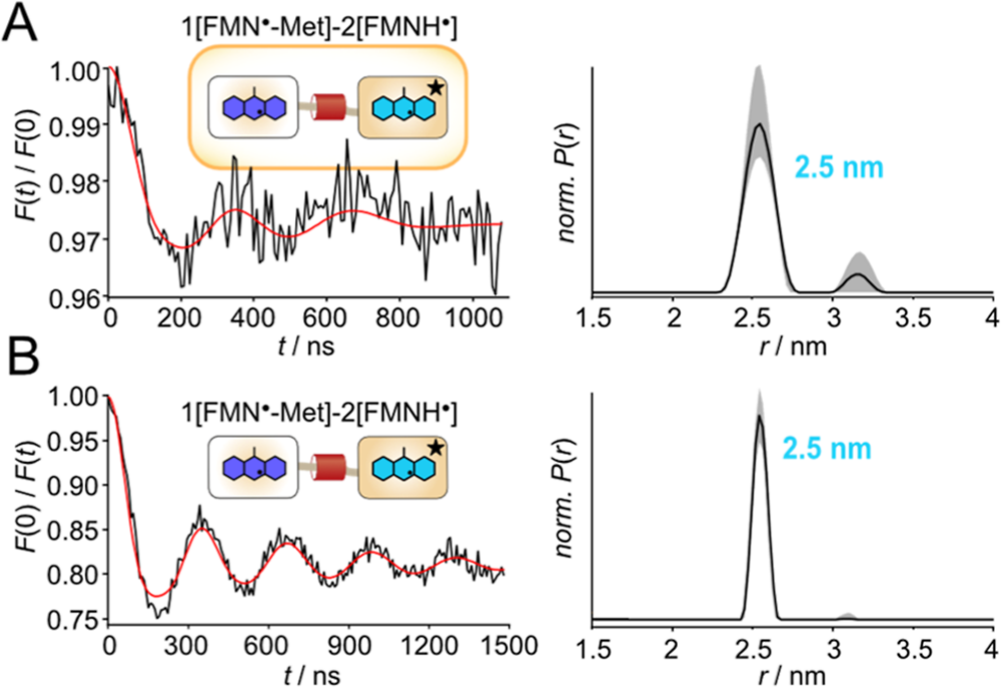
PELDOR spectroscopy of the 1­[FMN^·^-Met]-2­[FMNH^·^]^*^ fusion protein within cells (A) and in solution (B). Background-corrected time traces (black) and corresponding fits (red) are shown on the left; the corresponding distance distributions, as obtained by Tikhonov regularization with DeerAnalysis, are shown on the right. The gray shaded areas correspond to the error region from the Tikhonov validation.

**Table 2:** Comparison of FMN···FMN Distances Obtained from Dipolar Spectroscopy and from Structural Information^a^

FMN···FMN distance (PDS)/nm	FMN···FMN distance (crystal)/nm	PELDOR modulation depth	samples that show this distance
2.7 ± 0.2	2.6 ± 0.2	1.6^b^ and 2.5	1[FMN^·^-Met]
2.6 ± 0.2	(C4a-C4a)^27^	8.9	1[FMNH^·^]
2.5 ± 0.1	2.6 ± 0.2	17.8	1[FMN^·^-Met]-2[FMNH^·^]
2.5 ± 0.1	(C4a-C4a, this work)	11.4	1[FMN^·^-Met]···2[FMNH^·^]
2.5 ± 0.1		17.4	1[FMN^·^-Met]-2[FMNH^·^]^b^ (in vitro)
2.5 ± 0.1		2.4	1[FMN^·^-Met]-2[FMNH^·^]^b^ (in cell)
3.2 ± 0.3	3.2 ± 0.3	0.5	1[FMN^·^-Met]-2[^3^FMN]
3.2 ± 0.3	(C4a-N1)	0.4	1[FMN^·^-Met]···2[^3^FMN]
3.6 ± 0.2	3.4 ± 0.2	5.4	1[FMN^·^-Met]-2[FMN^·–^]
	(C4a-N5)		
5.1 ± 0.6		5.7	1[FMN^·^-Met]···2[FMN^·–^]
no defined distance		5.3	1[FMN^·–^]
		4.5	2[FMN^·–^]
		4.3	2[FMNH^·^]

a The respective atoms for which the distance was measured are indicated and correspond to the respective highest electron spin density. The FWHM values of the main distance peaks were assumed as uncertainties for the respective distances.

b The two values represent the modulation depths of 1­[FMN^·^-Met] and dark-adapted 1­[FMN^·^-Met], respectively (Figure 2A).

The lower signal-to-noise ratio for the in-cell measurement could be attributed to a lower concentration of FMN radicals, resulting from an incomplete FMN photoreduction in the cells. By comparing the intensities of the echo-detected field-swept (EDFS) spectra (Figure S13), we determined a protein concentration of 40 μM in the cell sample, which would lead to a radical concentration of 80 μM, if total reduction of all FMN cofactors is assumed. Nevertheless, the modulation depth and the signal-to-noise ratio are sufficiently high for a conclusive analysis, indicating that the photogenerated FMNH^·^ is stable under the reducing conditions of the cellular environment. In addition, the phase memory times are identical for both samples (Figure S14). The time trace analysis revealed a distance of 2.5 nm, analogously to the one obtained for 1­[FMN^·^-Met]-2­[FMNH^·^], with similar narrow distance distribution. The results of this experiment demonstrate that FMN radical generation is readily achievable in cells, that the concentration of both radicals is sufficient for PDS, and that the protein complex exhibits an identical conformation in cells and in solution.

### Structural and Electronic Information on the LOV Constructs

The question thus arises as to why the different combinations of paramagnetic FMN states result in different distances, and why the same result is not always obtained with distinct linker lengths. Two potential explanations for the observed results may be postulated. The observed distances between the FMN molecules may differ as a result of different electron spin density distributions of the different FMN species, particularly if the orientations of the two FMN radicals differ with respect to each other and between the samples. Accordingly, the spin density distributions of the three paramagnetic FMN states were calculated at the DFT level of theory (Figure 5A). The Mulliken spin density clearly shows that the electron spin density of the two FMN radical species is predominantly localized at the C4a and N5 atoms, whereas it is more uniformly distributed across the pteridine ring in the ^3^FMN state. However, it was determined that the orientation-dependent FMN distance, measured from the centers-of mass and taking into account different relative orientations and spin densities, can only vary by a maximum of 0.2 nm,^33^ which is significantly less than the experimentally observed distance differences.

**Figure 5:**
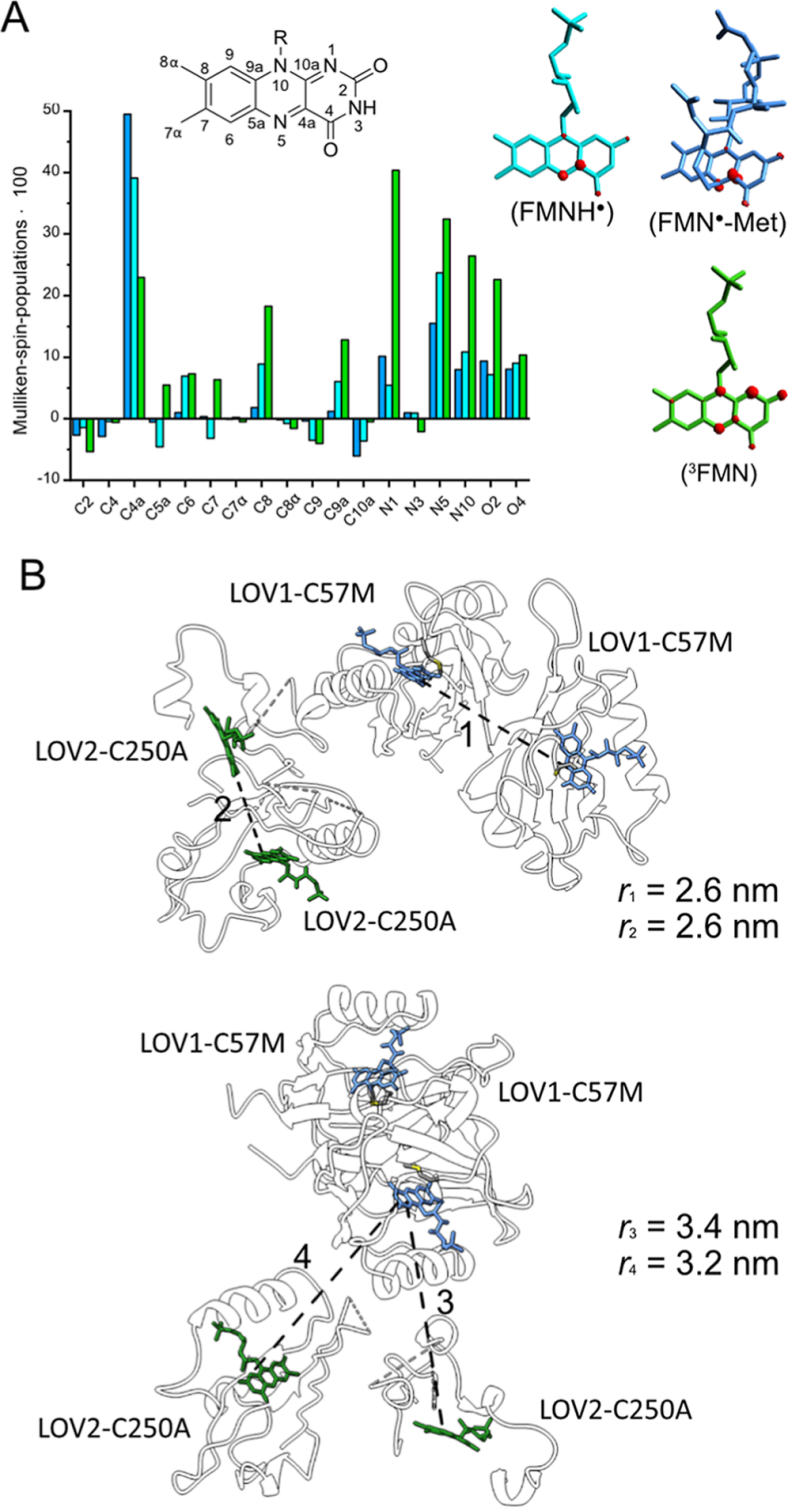
(A) Bar chart of the Mulliken spin populations on the individual atoms of the isoalloxazine moiety (color code as in Figure 1). The surface plot of the spin density of each FMN species is shown below. The spin densities were obtained from DFT calculations considering the amino acids within the radius of 4 Å radius of the respective FMN. (B) Structure the 1­[FMN^●^-Met]-2­[FMN] fusion protein. Two different orientations are depicted in the upper and lower panel. Selected FMN-FMN distances are highlighted.

Therefore, an alternative, second explanation appears to be probable. If different FMN redox states result in variations in conformation or degrees of multimerization, the distances between the two FMN domains would vary between samples. The LOV2-C250A domain and the LOV1-C57M-SL-LOV2-C250A fusion protein were therefore subjected to crystallization and examined using X-ray diffraction, with the aim of obtaining structural information from an independent method. The LOV2-C250A sample was crystallized in the dark, yielding a dimeric structure with highly similar interactions to those observed in LOV1-C57M. Accordingly the FMN···FMN distance is 2.6 nm (Figure S9). Prior to crystallization, the C57M-SL-LOV2-C250A fusion protein was subjected to preirradiation with blue light and reoxidation, ensuring that the two FMNs were in a consistent redox state comparable to that observed in ReLaserIMD measurements (FMN^·^-Met/FMN_ox_, Figure 1).

In the crystal structure (Figure 5B), only the LOV1 domains are fully resolved. The LOV2 domains are only partially resolved and the linkers are not resolved at all, presumably due to their flexibility, as the electron density at these positions is too low. Conversely, the position and orientation of the individual FMNs are well resolved and can be used for precise distance determination. Due to the higher electron density of the sulfur in the Met57 residue, the LOV1-C57 M domains could be unequivocally identified. The tertiary structure and the FMN binding position of the respective LOV domains are highly similar to previously published structures. Consequently, the focus will be on the quaternary structure and the respective FMN···FMN distances. Two fusion proteins are present per cell, comprising two LOV1 and two LOV2 domains. Of these, only one LOV1 domain is in direct contact with a LOV2 domain. The FMN···FMN distances of the LOV1 and LOV2 dimers are 2.6 nm each. Moreover, a multitude of alternative combinations of additional FMN···FMN distances exist, with the two shortest distances measuring 3.2 and 3.4 nm, respectively (Figure 5B).

### Comparison of Distances from PDS and Structural Information

The distances from the PDS measurements can be compared with the structural data based on the information above. When two LOV1 or two LOV2 single domains form homodimers, the FMN···FMN distance is consistently observed to be ∼2.6 nm, which is the case for the 1­[FMN^·^-Met] and 1­[FMNH^·^] single domains (Table 2). All LOV1-LOV2 fusion proteins in the FMN^·^-Met/FMNH^·^ redox states show very similar distances around 2.5 nm (Table 2), with small differences that can be attributed to the fact that the contact sites between LOV1 and LOV2 domains are slightly different. Nevertheless, both distances can be assigned to dimeric structures.

When the 1­[FMN^·^-Met]-2­[FMN] fusion protein is used for a ReLaserIMD experiment, only one distance of 3.2 nm is obtained, regardless of the linker length. This discrepancy could be due to the fact that both the short and long linker constructs are present as dimers, but the light-induced PDS experiment can only measure the distance between ^3^FMN and FMN^·^-Met. Because of the quasi-symmetry of the structure, the two distances (*r*_3_ and *r*_4_ in Figure 5B) are very similar, which may explain the acquisition of a single distance value.

It can be reasonably assumed that the quaternary structure of the FMN^·^-Met/FMN^·–^ samples remains unaltered following low temperature illumination. This raises the question of why only one FMN···FMN distance is observed, namely 3.6 nm for the short linker and around 5.0 nm for the long linker. It is unclear why the 2.6 nm distance, which is dominant in the other samples, cannot be observed despite the sufficient signal-to-noise ratio. Conversely, the sole discernible FMN···FMN distance of 2.5 nm in the (FMN^·^-Met/FMNH^·^) fusion protein is incongruous with the quaternary structure obtained from crystallography, as a 3.2 nm and other larger distances would manifest in the PELDOR time trace.

### Orientation Effects in LOV Fusion Proteins

To determine the correct distance distribution for each sample, the entire dipolar coupling tensor must be resolved in a PELDOR experiment, rather than only a subset due to orientation selection. In the presence of heavy orientation selection, Tikhonov regularization cannot be used to obtain accurate distance distributions. However, the main distance can always be extracted from the dominant perpendicular feature of the Pake pattern, which is typically observed experimentally.^34^ Therefore, the Pake patterns of all PELDOR time traces depicted in Figures 2–4 were calculated and the respective components of the dipolar tensor were extracted (Figures S18–S20). In most cases, only ω_⊥_ could be determined, and from that, the respective distances were calculated.^1a^ The differences between the distances obtained from the ω_⊥_ frequency and the regularization were within the margin of error (Table 2 and Figures S18–S20). Based on this, it can be concluded that orientation selection has no significant influence on the main distance observed in the distance distributions of any of the samples under the selected experimental conditions.

Additionally, the Pake patterns of selected measurements at different magnetic fields were compared. First, PELDOR measurements were conducted at three distinct magnetic fields using the 1­[FMN^·^-Met]-2­[FMNH^·^] sample. At all three positions, the prominent perpendicular component of the dipolar tensor was detected and exhibited an identical value of 2.95 MHz (Figure S10). Only if the detection was done at the maximum of the EPR signal, the parallel component with a value of 5.9 MHz was observed. Pake patterns were also compared for PELDOR experiments using the 1­[FMN^·^-Met]-2­[FMN^·–^] sample, and for the ReLaserIMD experiment (Figure S11). Here, both frequencies could be detected in the respective Pake pattern (Figure S12). This analysis suggests a stronger anisotropy for the flavin radicals than expected, probably due to the defined conformations of FMN in LOV domains and the slightly different hyperfine coupling constants of the different FMN species (Figure 5A). The extent of this effect varies depending on the construct used but must always be considered. While it is possible for some field positions to observe little to no modulations, distances obtained in this way for each investigated sample remain consistent, even in the cases where orientation selection heavily impacts on the recorded traces, thus it can be deduced that the difference in the distances observed for differently prepared redox states cannot originate from this.

Consequently, based on the results of the individual LOV1 and LOV2 constructs (Figure 2), it is likely that the two 1­[FMN^·^-Met]-2­[FMNH^·^] and 1­[FMN^·^-Met]···2­[FMNH^·^] constructs undergo a conformational change after light irradiation, leading to the formation of both LOV1-LOV1 and LOV2-LOV2 dimers. Thus, these constructs may have a different conformation than the one found in the crystal structure, which may explain the occurrence of only the 2.5 nm distance.

## Discussion

### LOV Domains as Spin Labels for Various PDS Experiments

This study investigated the potential of small flavin-dependent protein domains as adaptable spin labels. The generation of four distinct paramagnetic FMN redox states via an external stimulus, namely light, has been demonstrated (Figure 1). The stability of the respective FMN redox states can be modified by the selection of experimental conditions, including anoxic versus oxic environments, and illumination at RT or at low temperature. The lifetime of ^3^FMN is approximately a few milliseconds, after which it decays back to the FMN_ox_ ground state. FMN^·–^ and FMNH^·^ are long-term stable under anoxic conditions. Only the FMN^·^-Met redox state is stable at RT under oxic conditions. Therefore, paramagnetic FMN redox states in LOV domains can be utilized for all types of PDS experiments, and combinations of different FMN redox states can be prepared with ease and high yields. The use of different LOV-domain variants allows for straightforward and efficient orthogonal labeling, with all combinations of paramagnetic FMN states suitable for PDS. It is noteworthy that this is the first instance of ^3^FMN being successfully employed in light-induced PDS within a protein environment. This outcome significantly expands the scope of triplet spin labels.

To ascertain the stability, the fundamental EPR properties, in addition to the quaternary structure of the individual FMN redox states within LOV domains, the respective redox states were generated and examined by PDS. The quaternary structure of the LOV1 domain from remains a topic of debate. X-ray diffraction experiments yielded results indicating the presence of a monomer,^27^ whereas results from transient-grating spectroscopy suggested the existence of a mixture of monomers, dimers, and higher oligomers in the dark state. Furthermore, the degree of multimerization was observed to increase significantly following light exposure.^35^ Our findings indicate that the degree of oligomerization is contingent upon the LOV domain and the redox state of the FMN cofactor (Figure 2). While all investigated LOV2 domains are monomers, this is only true for LOV1 domains when irradiated at 80 K (Figure 2D). Otherwise, dimers (or higher oligomers) are formed (see also below).

All investigated proteins resulted in PELDOR time traces with sufficient modulation depths in the range between ∼0.5% for ReLaserIMD experiments and up to ∼17% for PELDOR experiments (Table 2). The results with the different constructs clearly show that we have a narrow distance distribution in most of the analyzed samples (Figures 3 and 4). One possible interpretation is that due to the short length of the linkers compared to the protein size, the conformational degrees of freedom of the LOV domains and especially those of the FMN cofactors are quite limited. Although this depends on the particular designed construct and cannot be generalized, our experiments show that narrow distance distributions can also be achieved with protein spin labels, even at a distance larger than 3 nm (Figure 3).

LOV domains represent one of the smallest cofactor-dependent protein domains, and thus the influence on the quaternary structure of the target protein is expected to be low. Despite the dimeric structure of the fusion protein, the experimental data did not always allow for the clarification of two questions: first, why only one distance could be obtained, and second, why the linker length only had an influence on the distance in the case of FMN^·–^. It should be noted that the primary objective of the study was not to elucidate the quaternary structure of the artificial LOV-LOV fusion proteins. Consequently, there will be no conclusive discussion of the undetectable distances. Nevertheless, the results of this study clearly demonstrate that different FMN radicals in LOV2 domains possess the requisite characteristics to serve as highly effective spin labels.

### LOV Domains as Versatile Spin Labels for In-Cell PDS

There are a few major challenges when performing in-cell EPR spectroscopy: first, the concentration of the protein to be analyzed is in most cases lower than in purified samples, which makes signal acquisition more difficult. Second, the published concepts are somewhat limited to specific organisms, which considerably restricts their applicability: to provide a natural environment including natural interaction partners for the POI, the same organism or cell line is mandatory. Furthermore, selective labeling is a limiting factor, as a strong background signal is generated if only one of the two necessary positions is labeled, or other proteins are labeled as side reactions.

The novel concept introduced here can provide solutions to all the aforementioned challenges: the usage of small, cofactor-dependent protein domains that can be attached to any protein of interest by cloning provides the possibility to use this system in any cells of interest; the labeling efficiency is solely dependent on the expression yield of the protein, which can be modulated by the usage of different expression plasmids, and only blue light is required for generation of the different paramagnetic flavin states, which is easy accessible and cheap. Thus, LOV domains can be used as spin labels in any cell system as long as an appropriate expression system and an illumination setup are available.

Our experimental data demonstrate that both the protein concentration and the radical generation are sufficient for successful analysis of in-cell PDS experiments. The distance measured in cells is comparable to the one in solution, therefore confirming that the quaternary structure of the LOV-LOV fusion protein does not change when examined in solution.

### Implications on LOV Domain Signal Transduction

While not the principal focus of this study, the results permit some conclusions to be drawn regarding the signal transduction of LOV domains. In wild-type (WT) LOV, the proposed mechanism for signal transduction from the FMN to the protein surface is as follows: the formation of the cysteinyl-4a-adduct results in a change in the hybridization of the C4a atom of the FMN from sp^2^ to sp^3^, accompanied by the simultaneous protonation of the adjacent FMN-N5 atom. The resulting conversion of the N5 position from a hydrogen bond acceptor to a donor is considered to be the primary trigger for a series of conformational and dynamic transitions, which may differ depending on the LOV receptor. These transitions include order/disorder transitions as well as oligomerization or other tertiary and quaternary structural changes.^36^

It was previously hypothesized that LOV domains can be photoreceptor active without forming the cysteinyl-4a-adduct, based on the results obtained from a VVD photoreceptor.^37^ Our comprehensive distance analysis of different LOV single domains containing different FMN radicals provides further insights into this matter: the obtained distances clearly demonstrate that LOV1 domains are invariably present as dimers when the protonated FMNH^·^ is present, but are monomers when illuminated at 80 K (Figure 3). This is due to the fact that significant protein movement, such as dimerization, is not feasible in a frozen state, and the protonation of FMN is inhibited at low temperature. Consequently, LOV1 exists as a monomer in the dark state, and a light-induced conformational change only occurs following the generation of FMNH^·^. In contrast, LOV2 domains do not undergo any conformational changes and remain monomeric across all experimental conditions (Figure 3).

Transient grating measurements of different LOV1 and LOV2 constructs yielded similar results, although these experiments were conducted with WT constructs in which the cysteinyl-4a-adduct was formed.^35^ This finding corroborates the hypothesis that FMNH^·^ induces an identical conformational change. This is likely due to the fact that the protonation of N5, which is essential for signal transduction, occurs in both FMN species, namely the cysteinyl-4a-adduct and the FMNH^·^. Additionally, an identical hydrogen bond is formed between N5 and the amino acid Gln120. It can be postulated that the protein will undergo further signal transduction, enabling dimerization in an identical manner. It should be noted that our work exclusively focuses on the core LOV domains, excluding additional helixes and kinase domains. This may potentially influence the observed conformational dynamics when analyzing different constructs.^35^ These findings align with the hypothesis that FMN radicals could activate LOV domains,^38^ although this phenomenon has only been observed in LOV1 domains of . It would be interesting to see whether this finding can be confirmed in other LOV domains.

### Conclusion and Future Perspectives

Although LOV domains are in principle very well suited as multifunctional genetically encoded spin labels, there are still some aspects that need to be addressed. One of these is that since the natural purpose of LOV domains is to sense and to transduce blue light, light-induced conformational changes or oligomer formation may occur, depending on the sample preparation or experiment, even if the essential cysteine is replaced. So far, only constructs using a 2­[FMN^·–^] domain have shown different and reliable distances for different linker lengths (Figure 3B). Currently, this issue limits applicability, but it can be solved by using other LOV domains that are known to remain monomeric during radical formation. A suitable candidate would be, for example, the iLOV protein designed for fluorescence imaging.^39^

It is essential for the stability of FMN radicals in cells that they are effectively shielded from the environment to keep the radical away from other redox-active substances. Therefore, such flavin-based protein spin labels need to be of a certain size. The molar mass of the LOV domains used in this study is less than 12 kDa (Figure 1C), which is light for a protein, but significantly heavier than organic spin labels. This difference could, in principle, have disadvantages in terms of labeling efficiency and accuracy when determining long distances. Figure 2A shows that two LOV domains in direct contact have a FMN···FMN distance of 2.7 nm, so the useable distance range is reduced compared to other (organic) spin labels. The additional distance between the protein surface and FMN makes it more difficult to accurately determine larger distances (>5.5 nm). However, the extent of this difficulty depends on the position of the labels and the change in conformation, so it cannot be generalized. On the other hand, the additional distance may also have positive effects. For example, if the actual distance of interest is less than 1.5 nm, and is therefore too short for PDS, the additional distance may allow it to be used.

Proteins that contain a fluorophore such as GFP, which has a molecular mass of ∼25 kDa, have been widely used in various fluorescence experiments for some time, and for many of those experiments the advantages of a genetically encoded label seem to outweigh its disadvantages.^40^ We believe our protein-based spin labels to have an analogous potential. In addition, the size of the LOV domains could be further reduced by omitting amino acids that are not essential for folding and FMN binding. Sequence alignments comparing the characterized LOV domains suggest that a reduction of up to 15% is possible.

For the use of LOV domains as triplet spin labels, FMN photoreduction at low temperature should be prevented. This can be achieved either by introducing point mutations at the potential electron donor positions, by incorporating chemically modified FMN derivatives with different redox properties,^41^ or by using other small flavoproteins such as BLUF domains, which have been shown to effectively form flavin triplet states and are not easy to be photoreduced.^42^

The initial application of distance measurements in model proteins is a logical progression; for example, a protein that undergoes a conformational change in the presence of a substrate would provide significant insight into the benefits of using protein spin labels. The basic methodology for using LOV spin labels is outlined in Scheme 1. As with other genetically encoded labels, N- and C-terminal LOV domains can be easily attached to the POI at the DNA level, and the fusion protein is expressed in the organism of interest. The protein can then be purified and the required FMN radicals produced, or this step can be performed directly in cells. The selected PDS experiments can then be performed and analyzed. Such experiments on a model protein, for which the structure is known and conformational changes have already been studied, could also shed light on the orientation selection that occurred in the PELDOR results presented here. Orientation effects in different combinations of FMN radicals could be quantified, perhaps to be used in the future to obtain not only distance but also orientation information even at moderately high magnetic field strengths (all measurements were performed at Q-band frequencies). We look forward to seeing the first results with LOV spin labels, both in vitro and in intact cells, very soon.

**Scheme 1:**
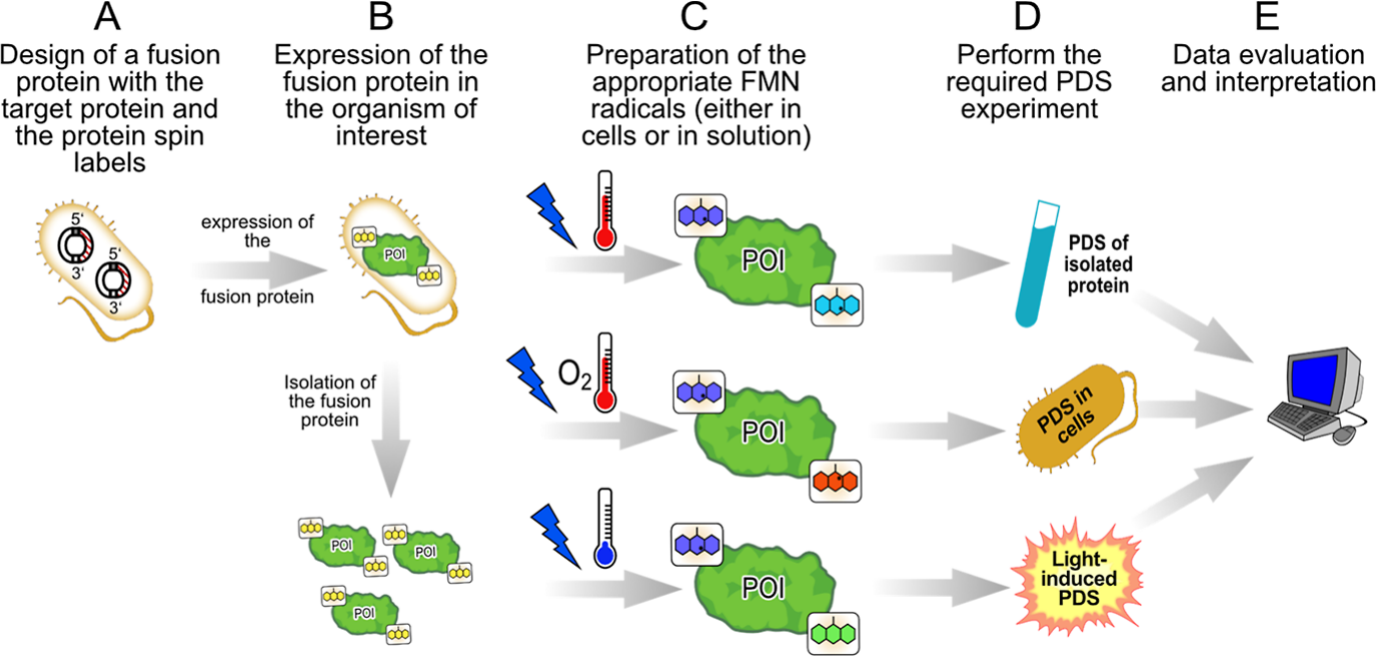
Usage of LOV Domains as Versatile Protein Spin Labels^a^

a The designated LOV domains are attached to the N- and C-termini of the protein of interest at the DNA level (A) and the resulting fusion protein is produced in the selected cells (B). Isolation of the protein is optional. Subsequently, the two LOV domains are converted to the required oxidation states (FMN_ox_/FMN^·^-Met/FMNH^·^/FMN^·–^) (C). All types of PDS experiments can now be performed with the fusion protein (D), and subsequently analyzed (E).

## Experimental Section

### Cloning

The amino acid sequences of the LOV domains were taken from^43^ and back translated to the respective DNA sequences using the open source EMBOSS software.^44^ The base triplets encoding the Cys residues that are involved in light-driven adduct formation with the FMN cofactor were replaced by base triplets encoding for Met (position 57 in LOV1) and Ala (position 250 in LOV2), respectively. The amino acid sequences for the helical linkers were taken from^24a^ and back translated into the DNA sequence as described above. *Eco*RI and *Hin*dIII restriction sites were added to the 5′- and 3′-termini of the linker sequence, respectively, to allow subsequent cloning steps. The gene encoding the LOV1-LOV2 construct fused by the short linker was codon optimized for heterologous gene expression in and cloned into the pET28a­(+) vector using the NdeI and BlpI restriction sites by the GenScript company. The construct included a sequence for a N-terminal-(His)­6-tag for protein purification. The dsDNA sequence encoding the long linker was purchased from Biocat (Heidelberg) and cloned into the target vector using the *Eco*RI and *Hin*dIII restriction sites.

The DNA encoding the gene for the WT LOV1 single domain was purchased from Thermo Fisher Scientific and cloned into the pET28a­(+) vector using the *Hin*dIII and *Bam*HI restriction sites. The mutations of the conserved Cys C57 to Ala or Met were introduced by site-directed mutagenesis using the primers given in Table S1. The gene encoding the LOV2-C250A single mutant was cloned from the plasmid into the pET28a­(+) vector using the *Hin*dIII and BlpI restriction sites. The mutation W291F in the LOV1-LOV2 fusion protein with the short linker was introduced by site-directed mutagenesis using the primer pair given in Table S1. The DNA sequences of all constructs gained from cloning procedures were verified by Sanger sequencing (Eurofins Genomics) using the sequencing primer listed in Table S1.

### Protein Production and Purification

All plasmids were transformed into chemical competent SoluBL21 bacteria by using the heat shock method. LB medium containing Kanamycine (50 μg/mL) was inoculated with an overnight grown liquid cell culture (1% v/v). The cultures were cultivated at 140 rpm and 37 °C until the OD_600_ reached a value of 0.5. The gene expression was induced by adding IPTG (0.5 mM). Cells were incubated at 18 °C overnight, harvested by centrifugation (4 °C, 7000 rpm, 15 min) and shock-frozen in liquid nitrogen. The frozen cell pellet was resuspended in buffer A (50 mM HEPES, pH = 7.0, 100 mM NaCl, 10% glycerol) by stirring at 4 °C. PMSF (100 μM) and a spatula tip of DNaseI were added to the suspension and the cells were mechanically disrupted at 1100 bar in two cycles using a Microfluidizer (Microfluidics). The raw extract was centrifuged (18,000 rpm, 4 °C, 30 min) and the supernatant was loaded on a 5 mL HisTrap column (GE Healthcare) by using a ÄKTAgo chromatography system (GE Healthcare). The column was washed with binding buffer (50 mM HEPES, pH = 7.0, 200 mM NaCl, 10% glycerol, 20 mM imidazole) until the absorption at 280 nm reached a constant level. The protein was eluted by applying a linear Imidazole gradient using elution buffer (50 mM HEPES, pH = 7.0, 200 mM NaCl, 10% glycerol, 500 mM imidazole). The yellow fractions containing the LOV proteins were concentrated by centrifugation in filter units (Amicon Ultra, 10 kDa MWCO, 4000 rpm, 4 °C). Imidazole was removed by diluting the sample with buffer B (50 mM HEPES, pH = 7.0, 100 mM NaCl, 20% Glycerol) and concentrating it to the initial volume as described above in three cycles. The purity was analyzed by SDS PAGE and was sufficient for all proteins, so no further purification steps were applied.

### UV–vis Spectroscopy

UV–vis spectra were recorded to determine the protein concentration and to study the photochemical processes of the LOV domains upon blue-light irradiation. The spectra were recorded under continuous cooling to 4 °C (Julabo F20 pump and Julabo HC water thermostat) of the sample cuvette (Hellma 105.250-QS). The samples were irradiated with a blue light LED (M455L4, 455 nm, LED power 95 mW, Thorlabs). The concentration of flavoproteins was determined by using the free FMN’s absorption coefficient (12,200 M^–1^ cm^–1^).^45^

### EPR Sample Preparation

Glycerol was added to the protein samples, initially dissolved in buffer B, to a final amount of 60% in order to form a glassy matrix in the frozen state. The protein concentration was set to ∼200 μM. Samples were filled into quartz glass tubes (1 mm inner diameter) and irradiated with a blue-light LED as described above until the sample turned blue. For experiments with both FMNs in the radical state, the sample was shock frozen immediately after irradiation. If FMN in the LOV2 domain needed to be in its oxidized state, the sample was incubated on air overnight at 4 °C and then shock frozen in liquid nitrogen.

For in-cell EPR experiments the SoluBL21 cells were washed by centrifugation (4000 rpm, 4 °C) and resuspension in buffer B in three cycles. Finally, the cells were resuspended in buffer B to yield an OD_600_ of ∼80. The cell suspension was transferred into a Q-band EPR tube and irradiated with a blue-light LED. In order to monitor the formation of FMN radicals, cw-EPR spectra were acquired at 100 K (Figure S8). In-cell protein concentration was determined by measuring echo detected field swept spectra of proteins in cells and in vitro using identical experimental parameters (Figure S13). The concentration of the sample in aqueous solution was calculated to be 165 μM by absorption spectroscopy. From the echo intensities, the protein concentration in the cell sample was calculated to be approximately 40 μM. In order to examine the cell vitality before and after the irradiation procedure the sample was diluted to a OD_600_ of 2 and illuminated under the same conditions as for the EPR preparation. The cells were observed before and after blue-light exposure under a light microscope (Primo Star, Carl Zeiss).

### cw-EPR

All cw-EPR measurements were carried out on an EMX-Nano (Bruker) spectrometer at X-band (ν_MW_ ≈ 9.7 GHz) frequencies. The samples were filled into quartz glass tubes (Ilmasil PS, Qsil) with an inner diameter of 1 mm and placed in a regular X-band tube (inner diameter = 3 mm). The temperature was set to 100 K using a nitrogen gas flow cryostat (variable temperature accessory, Bruker). Sample-specific parameters are given in the respective spectra.

### Transient EPR

Transient X-band EPR spectra were measured in an ElexSys EPR spectrometer (E580, Bruker). A critically coupled, dielectric MD5-W1 resonator (model: ER4118X, Bruker) was used. The microwaves (MWs) were generated in an XFTu MW bridge (Bruker) and its power was set to 1.5 mW. All trEPR measurements were performed at 80 K, with the cavity cooled by a gas flow cryostat with nitrogen (CF935, Oxford Instruments) and the temperature adjusted by a PID controller (ITC4, Oxford Instruments). A digital delay generator (Model DG545, Stanford Research Systems) was used to position the laser pulse and trigger the spectrometer. The light excitation was performed by an LED-pumped laser with integrated OPO (NT 230-50, EKSPLA). The excitation wavelength was 460 nm for all measurements and the shot frequency of the laser was set to 25 Hz. For transient EPR measurements, the sample was excited through the optical window of the resonator. The emission energy was measured with a pyrolelectric detector (QE25LP-S-MB-QED-D0, genteceo) and adjusted to 3.5 ± 0.3 mJ/pulse with a λ/2 wave plate. The transient EPR signal was amplified by a low-noise preamplifier (Model SR560, Stanford Research Systems).

### Pulsed EPR Spectroscopy

Pulsed EPR measurements were performed on the same setup as described in the transient EPR section. All spectra were measured at Q-band frequencies using a dielectric ring Q-band resonator (model EN5107D2, Bruker). The MWs were generated with a XFTu MW bridge and converted to Q-band frequency (ν_MW_ ≈ 34 GHz, Super QFTu-EPR Bridge, Bruker). The MW pulses were amplified via a 50 W solid-state amplifier (AMPQ34 GHz, Bruker). This amplifier was installed during this project, so the MW power and pulse lengths had to be readjusted (the attenuation was set to 0 dB before and to 8–10 dB after the installation for a π-pulse length of 32 ns, respectively). Apart from the ReLaserIMD experiments, which were set up at 20 K, the temperature was set to 80 K. With the exception of the ReLaserIMD and ENDOR experiments, the resonator was always overcoupled to enable the broadest possible excitation bandwidth. EDFS spectra were recorded by applying a standard Hahn-echo sequence with τ = 400 ns. Phase memory time (*T*_M_) measurements were performed as single-point acquisitions at the maximum of the radical spectrum by increasing the interpulse delay in 8 ns increments. The initial τ was set to 180 ns. For light-induced EDFS spectra the sample was excited with the same laser setup described in the transient EPR section and the delay after flash (DAF) was set to 1 μs. As the formation of a FMN anionic radical was observed during the light-excitation of the samples at low temperature, the laser beam was coupled to an optical fiber (outer diameter = 0.9 mm, Thorlabs) in order to irradiate the whole sample and enable efficient radical formation.

### PELDOR Spectroscopy

Optimal magnetic field positions for excitation and detection pulses were determined before the actual PELDOR experiments. In measurements with FMNH^·^ and FMNH-Met^·^ cofactors, the pump pulse was positioned at the maximum of the EDFS spectrum, and detection was performed at 42–50 MHz high-field shifted. Measurements with FMN^·–^ showed a pronounced orientation selection, so the pump- and observer-pulse positions were optimized (Figure S12): maximum modulations were obtained when the observer pulse was positioned at the maximum of the EDFS spectrum and the pump pulse was high field shifted by 45 MHz.

A standard π/2 – τ_1_ – π – *t* – π _Pump_ – (τ_1_ + τ_2_ – *t*) – π – τ_2_ – *refocused echo* sequence was used to acquire PELDOR time traces. τ_1_ was set to 400 ns and τ_2_ was adjusted with respect to the desired length of the dipolar trace and the signal-to-noise ratio. A 2-step phase cycle was used to eliminate trigger offsets. The shot repetition time was set to at least 2.5 ms.

### ReLaserIMD Spectroscopy

The resonator was coupled in order to maximize the MW power inside the cavity. The ReLaserIMD sequence were recorded on the maximum of the FMNs radical spectrum. The following pulse sequence has been used: π/2 – τ_1_ – π – *t* – laser pulse – (τ_1_ + τ_2_ – *t*) – π – τ_2_ – *refocused echo*. τ_2_ was set to 400 ns and τ_1_ set to 1400 ns (8 ns increment) for the short linker construct and to 2000 ns (16 ns increment) for the long linker construct, respectively. For ReLaserIMD the sample was excited with the same laser setup described in the transient EPR section. The position of the laser pulse within the MW sequence was adjusted by a delay generator (Scientific Instruments). The MW pulse sequence was moved with respect to the position of the laser pulse in the experiment. A 2-step phase cycle was applied in order to eliminate trigger offsets.

### Davies ENDOR Spectroscopy

Radio frequency (RF) pulses for ENDOR measurements were generated by an RF generator (Dice II RF Controller, Bruker) and amplified with a 250 W amplifier (250A250A, Amplifier Research). The experiment was performed at the maximum of the EDFS spectrum by using the following pulse sequence: π – *T* – RF – *T* – π/2 – τ – π – τ – *inverted echo*. In order to excite selective electron spin and nuclear spin transitions, the resonator was coupled slightly and long pulses were used (*t*_π_ = 120 ns, *t*_RF_ = 13 μs). The pulse intervals *T* and τ were 1000 and 400 ns, respectively. The shot repetition rate was set to 18 ms to ensure the return of the magnetization vector to the equilibrium state. The ENDOR spectrum was recorded by integrating over the inverted echo as a function of the stochastically varied RF.

### Analysis of PDS Experiments

Raw time traces from PDS (Figures S15–S17) were analyzed using either the Matlab program DeerAnalysis (version 2022)^46^ or the Python program GloPel (version 1.0.1).^47^ The time axis of the ReLaserIMD time traces was cut at *t* = τ_1_. After automatic phase correction, a homogeneous background was subtracted from the experimental spectrum (*d* = 3). The distance distribution from the time trace was obtained by a Tikhonov regularization. The regularization parameter α was chosen by the left-corner criterion (different L-curves for selected time traces are shown as Figure S21). The resulting distance distribution was validated by adding white noise with a factor of 1.2–1.5 (10 steps) to the experimental form factor and varying the starting time of the background function (11–25 steps). The signal-to-noise ratio was calculated by dividing the modulation depth by the root mean square deviation of the imaginary part after phase correction.

### Crystallography and X-ray Diffraction

The LOV fusion protein was crystallized in 20% PEG 3350, 0.1 M BIS-TRIS, pH 6.5, and 0.1 M ammoniumsulfate at a concentration of 5 mg/mL. Protein crystals appeared within 2 days and were cryo-protected in 10% 2,3-butanediol prior to flash-freezing in liquid nitrogen. A data set was recorded at beamline X06DA (PXIII) at the Swiss Light Source of the PSI in Villigen, Switzerland. The data were processed using autoPROC.^48^ The phase problem was solved by using phaser^49^ from the ccp4 suite^50^ with the PHOT-LOV1 domain from (PDB: 1N9L) as initial search model. Iterative refinement in real space and reciprocal space was carried out in COOT^51^ and BUSTER,^52^ respectively.

### DFT Calculations

Only the hydrogen positions were optimized in the FMN structures taken from the respective domains in the crystallographic structure of the linker construct including the relevant residues for LOV1-C57M: Met57, Arg59 and Arg75 and for LOV2-C250A: Arg210 and Arg226. These geometry optimizations and spin density calculations were performed at the B3LYP/6–311G­(d,p) level of theory as implemented in Gaussian 16 (revision B.01).

## Supplementary Material



## Notes

Additional EPR and optical spectra, crystal structures of LOV domains and a list of primers used (PDF)

## ∥

Department of Chemistry, Photon Science Institute and The National Research Facility for Electron Paramagnetic Resonance, University of Manchester, Oxford Road, Manchester M13 9PL, UK

## Abbreviations

continuous wave: cw
electron paramagnetic resonance: EPR
echo-detected field swept: EDFS
electron–nuclear double resonance spectroscopy: ENDOR
flavin mononucleotide: FMN
laser-induced magnetic dipole: LaserIMD
light-oxygen-voltage: LOV
microwave: MW
protein of interest: POI
light-oxygen-voltage: LOV
pulsed dipolar spectroscopy: PDS
pulsed electron–electron-double-resonance: PELDOR
radiofrequency: RF
refocused LaserIMD: ReLaserIMD
room temperature: RT
wild-type: WT
