# Supplementary material for: Small Protein Domains as Potential Spin Labels for In Vitro, Cellular, and Light-Induced Dipolar EPR Spectroscopy
Source: J Am Chem Soc. 2025 Jun 23;147(26):22473–87. doi: 10.1021/jacs.5c01875 (PMC12232321; doi:10.1021/jacs.5c01875)
Supplement: Supplementary file 1 [file ja5c01875_si_001.pdf]

Small Protein Domains as Potential Spin Labels for In Vitro, Cellular, and Light-Induced Dipolar EPR Spectroscopy

Andreas Günter[†], Susanna Ciuti[‡], Lukas Denkhaus[§], Anna Sappler[†], Laura Orian[‡], Stefan Gerhardt[§], Oliver Einsle[§], Stefan Weber[†], Marilena Di Valentin[‡], and Erik Schleicher*[†]

[†] Institut für Physikalische Chemie, Albert-Ludwigs-Universität Freiburg, Albertstr. 21, 79104 Freiburg, Germany

[‡] Dipartimento di Scienze Chimiche, Università di Padova, via Marzolo 1, 35131 Padova, Italy

[§] Institut für Biochemie, Albert-Ludwigs-Universität Freiburg, Albertstr. 21, 79104 Freiburg, Germany

Supporting Information

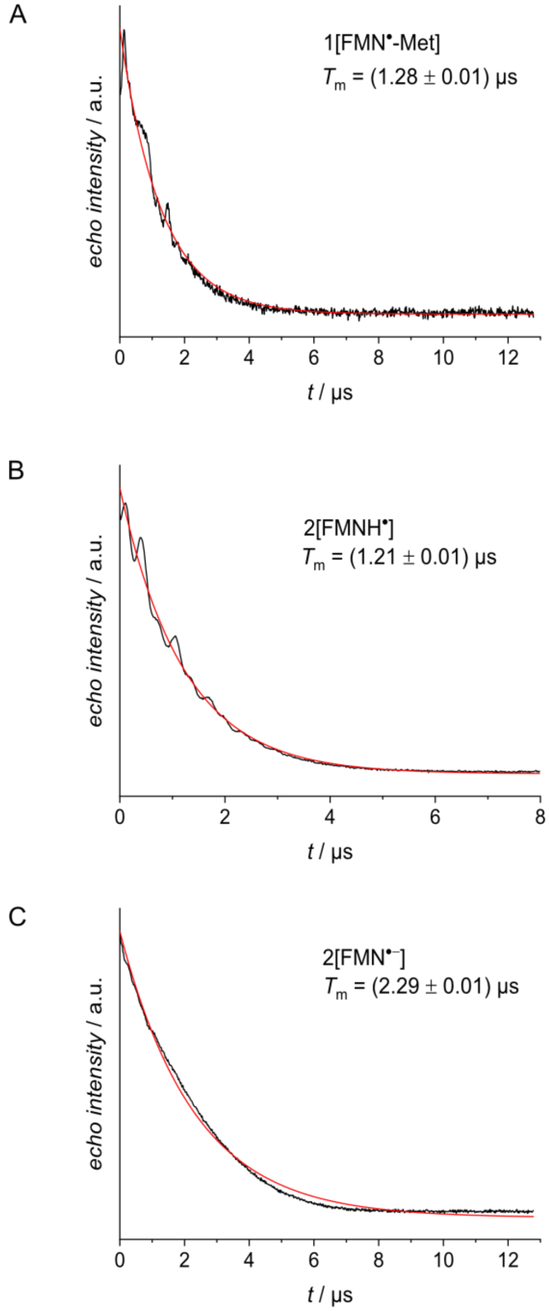

Figure S1. Experimental echo decay traces (black) and corresponding monoexponential fits (red) for the T_M determination of different FMN radicals in LOV domains. A) LOV1-C57M(FMN•-Met), B) LOV2-C250A(FMNH•) and C) LOV2-C250A(FMN•–). The measurements were performed as single-point acquisitions at the maximum of each radical spectrum by increasing the interpulse delay in 8 ns increments. The initial τ was set to 180 ns.

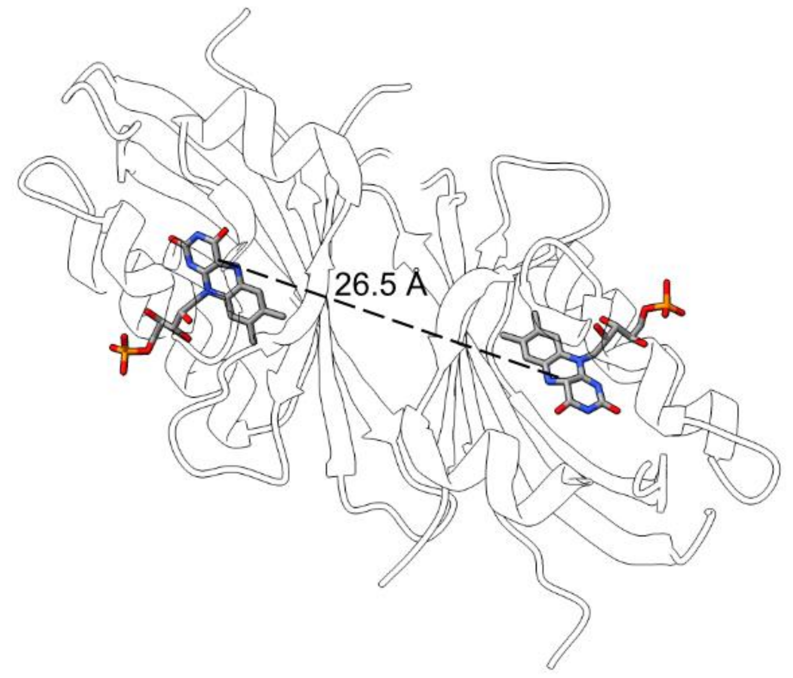

Figure S2. Dimeric structure of the wildtype *Cr*LOV1 domain in the lit state, calculated by the PISA software (based on the PDB number 1N9O, biological assembly 3).[1] The shown distance was measured between the two C4a atoms of the FMN moieties.

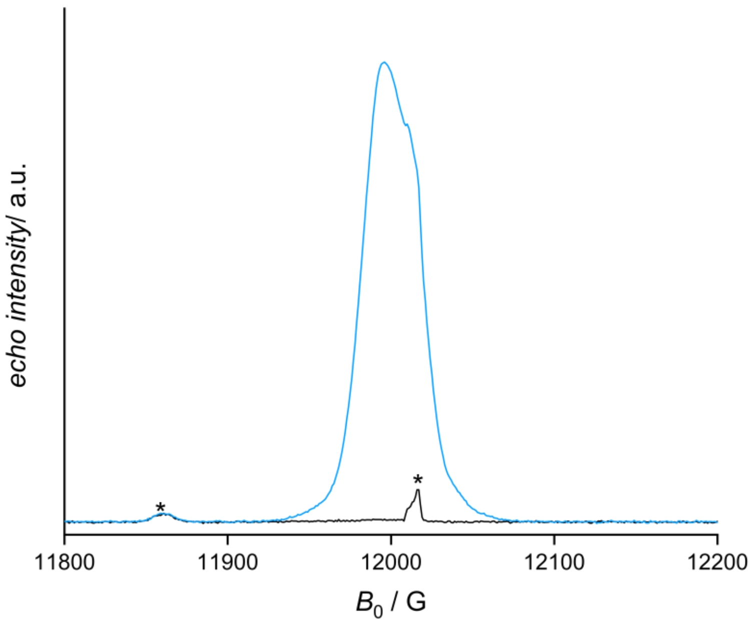

Figure S3. EDFS spectra of the LOV2-C250A mutant in the dark state (black) and after 3 min laser irradiation (460 nm) at 80 K (light blue). The sample was irradiated from the top via an optical fiber. Resonator background is labeled by asterisks.

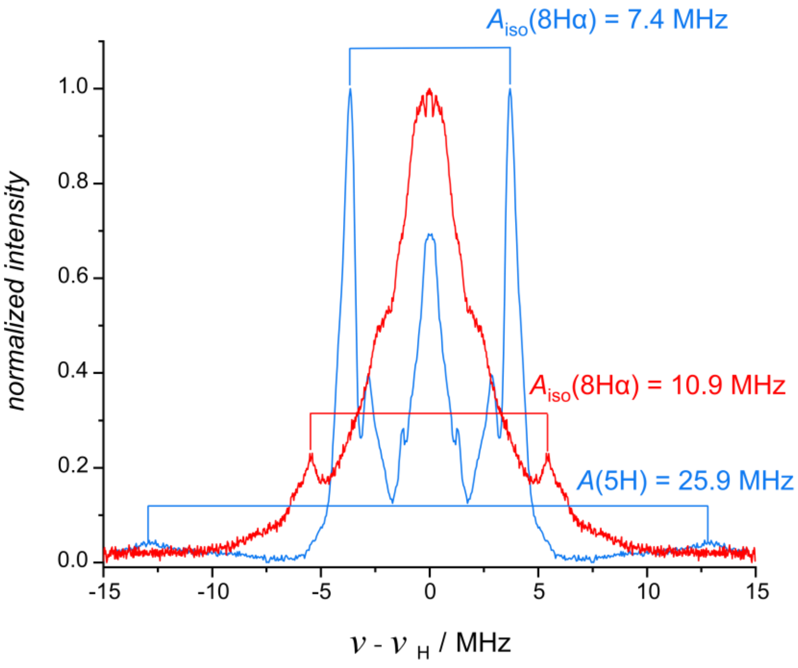

Figure S4. ^{1}H-Davies ENDOR spectra of LOV2-C250A in the $FMNH^{\bullet}$ state (red spectrum) and in the $FMN^{\bullet -}$ state (blue spectrum). Both ENDOR spectra were recorded at the maximum of the EDFS spectra.

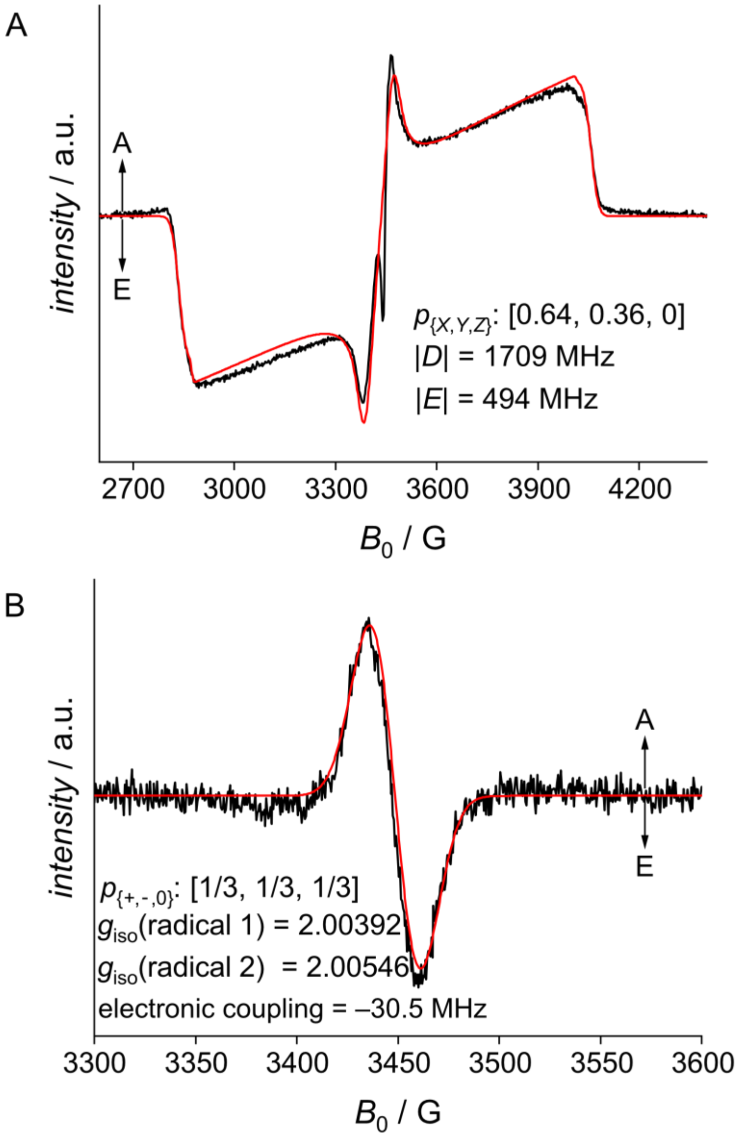

Figure S5. X-band transient EPR spectra (black) and their spectral simulations [2] (red) of a LOV2-C250A sample recorded 1.8 µs (A) and 14.4 µs (B) after light excitation, respectively.

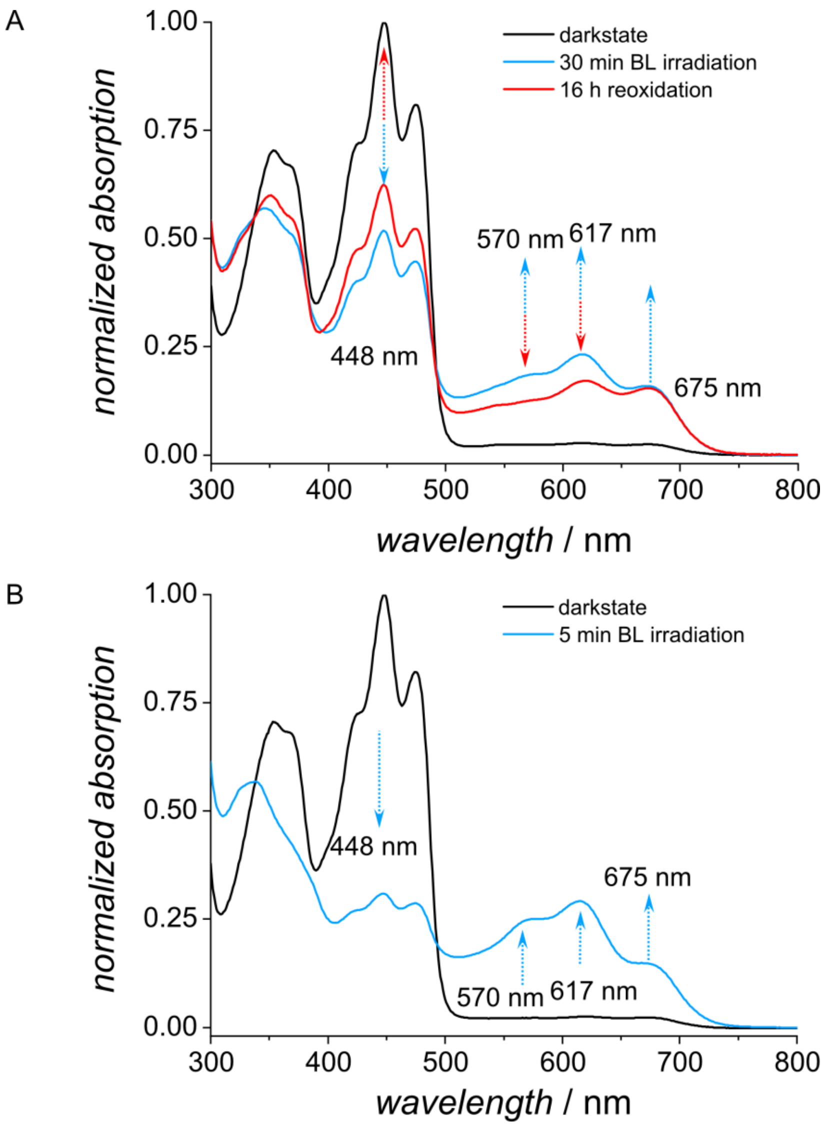

Figure S6. UV-vis spectra of the LOV fusion proteins 1[FMN-Met]-2[FMN] (A) and 1[FMN-Met]---2[FMN] (B). The arrows indicate the increase or decrease of the respective absorption band during the photoreduction (blue) or reoxidation (red) processes. The LED power was set to 30 mW in A) and to 95 mW in B).

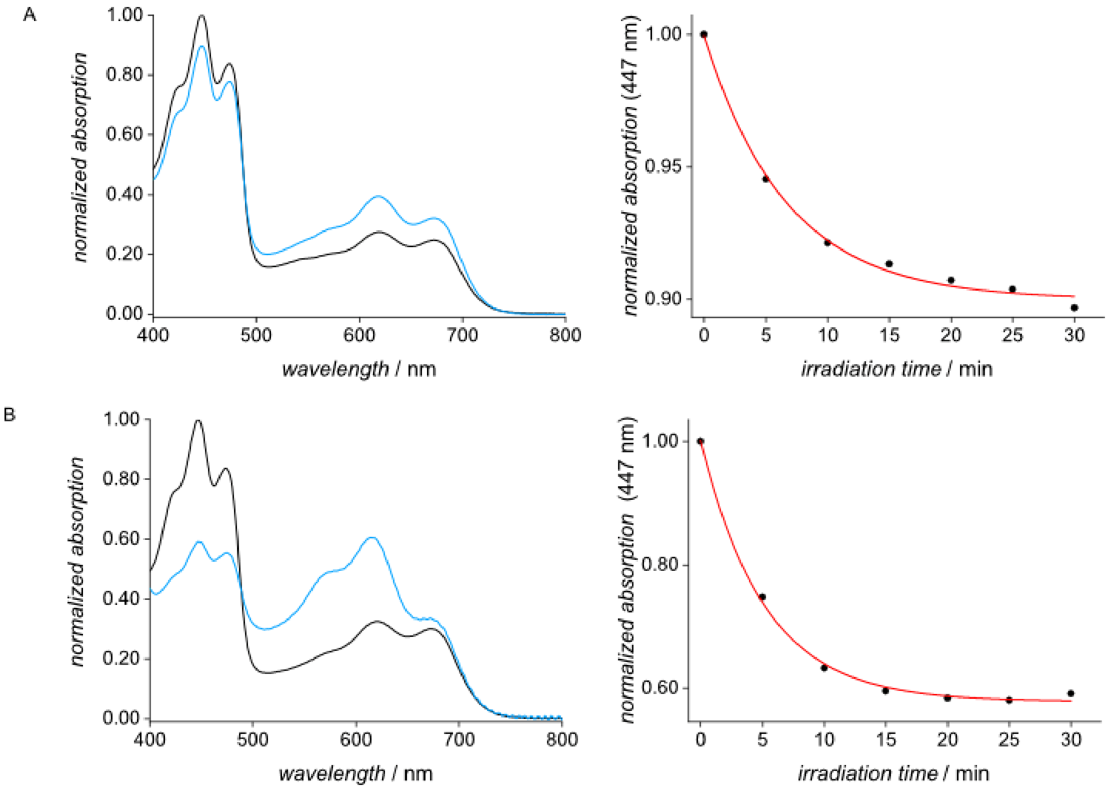

Figure S7. Left panels: UV-vis spectra of the fusion proteins 1[FMN$^\bullet$-Met]-2[FMN_{ox}] (A) and 1[FMN$^\bullet$-Met]-2[FMN_{ox}]* (B) in the dark (black) and after blue light illumination (light blue). The samples were pre-illuminated and reoxidized before the experiments to eliminate the influence of FMN$^\bullet$-Met bound to LOV1. Right panels: Photoreduction kinetics of the fusion proteins. The determined rate constants are k_{W291} = (0.149 ± 0.013) min^{-1} and k_{W291F} = (0.193 ± 0.011) min^{-1}.

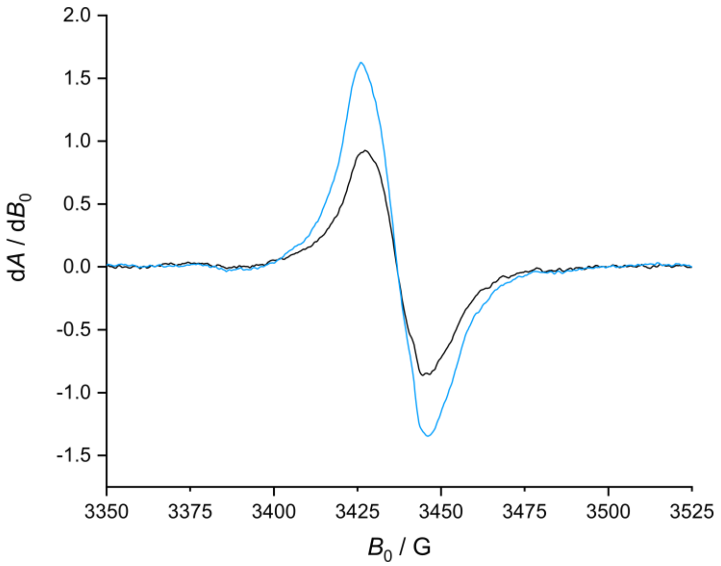

Figure S8. cw-EPR spectra of SoluBL21 *E. coli* cells containing the 1[FMN$^{\bullet}$-Met]-2[FMNH$^{\bullet}$]* fusion protein in the dark (black) and after 60 min of blue light illumination (light blue). The spectra were recorded at 100 K, a modulation amplitude of 3 G, 0.31 mW microwave power and 10 accumulations.

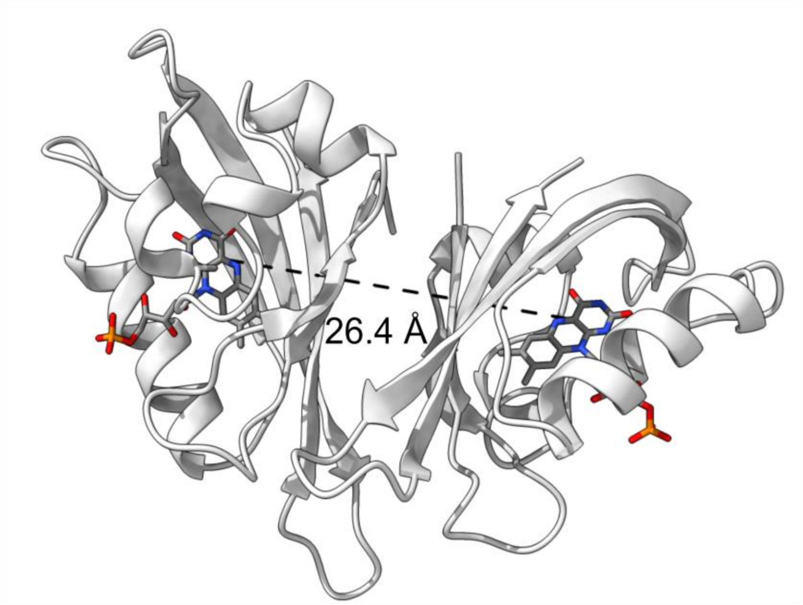

Figure S9. Crystal structure of the *Cr*LOV2-C250A mutant in the dark state. The shown distance was measured between the C4a atoms of the FMN moieties. The conditions for crystal growth and x-ray diffraction are given in the experimental section of the main manuscript.

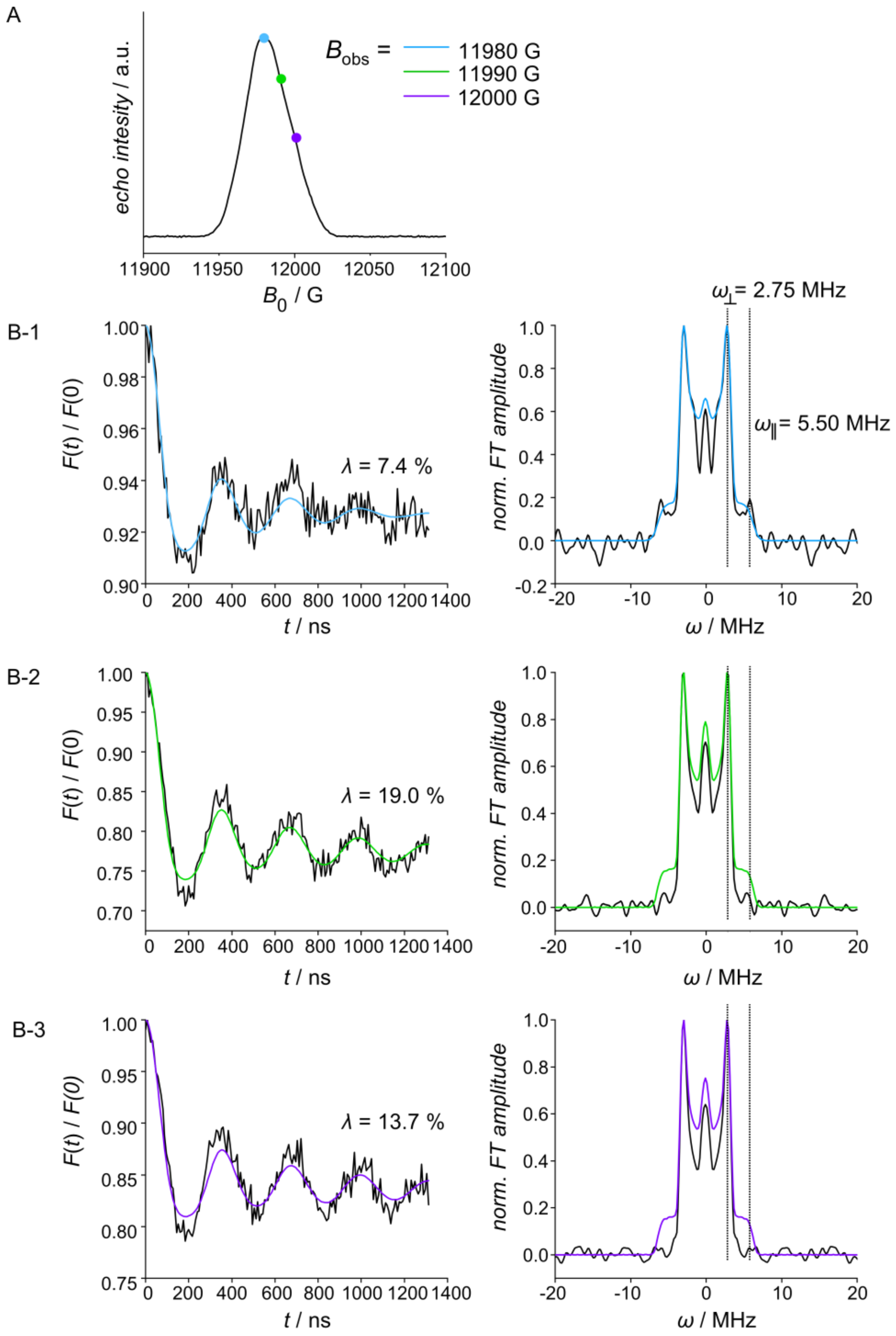

Figure S10. A) Echo-detected field-sweeped (EDFS) EPR spectrum of a 1[FMN•-Met]-2[FMNH•] sample with outlined observer positions that were used for PELDOR measurements on LOV proteins containing neutral FMN radicals. The pump field was set to B_{Pump}= B_{Obs} – 17 G. B). Dipolar time traces and their Tikhonov-fits (left panel) and the respective Fourier-transformed frequency spectra (right panel). *λ* is the modulation depth.

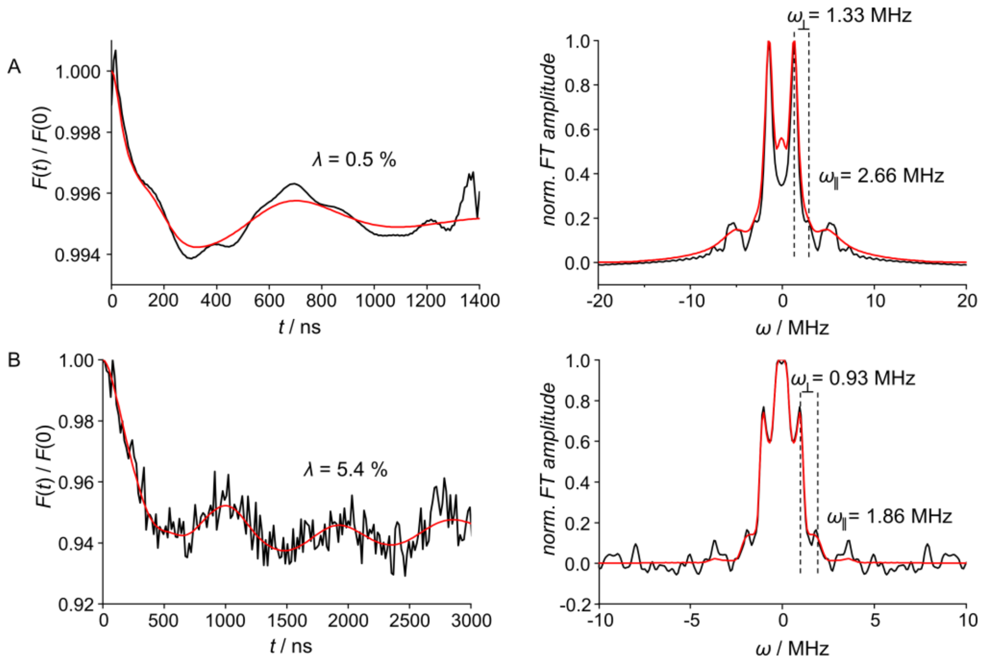

Figure S11. Dipolar time traces, Tikhonov fits and the corresponding frequency spectra of the 1[FMN-Met]-2[FMN] construct. A) ReLaserIMD experiment on the 1[FMN$^{\bullet}$-Met]-2[^{3}FMN] sample. The high frequency noise of unknown origin was removed by applying a long pass filter (see Figure 3 in the main manuscript). B) PELDOR experiment on the 1[FMN$^{\bullet}$-Met]-2[FMN$^{\bullet-}$] sample.

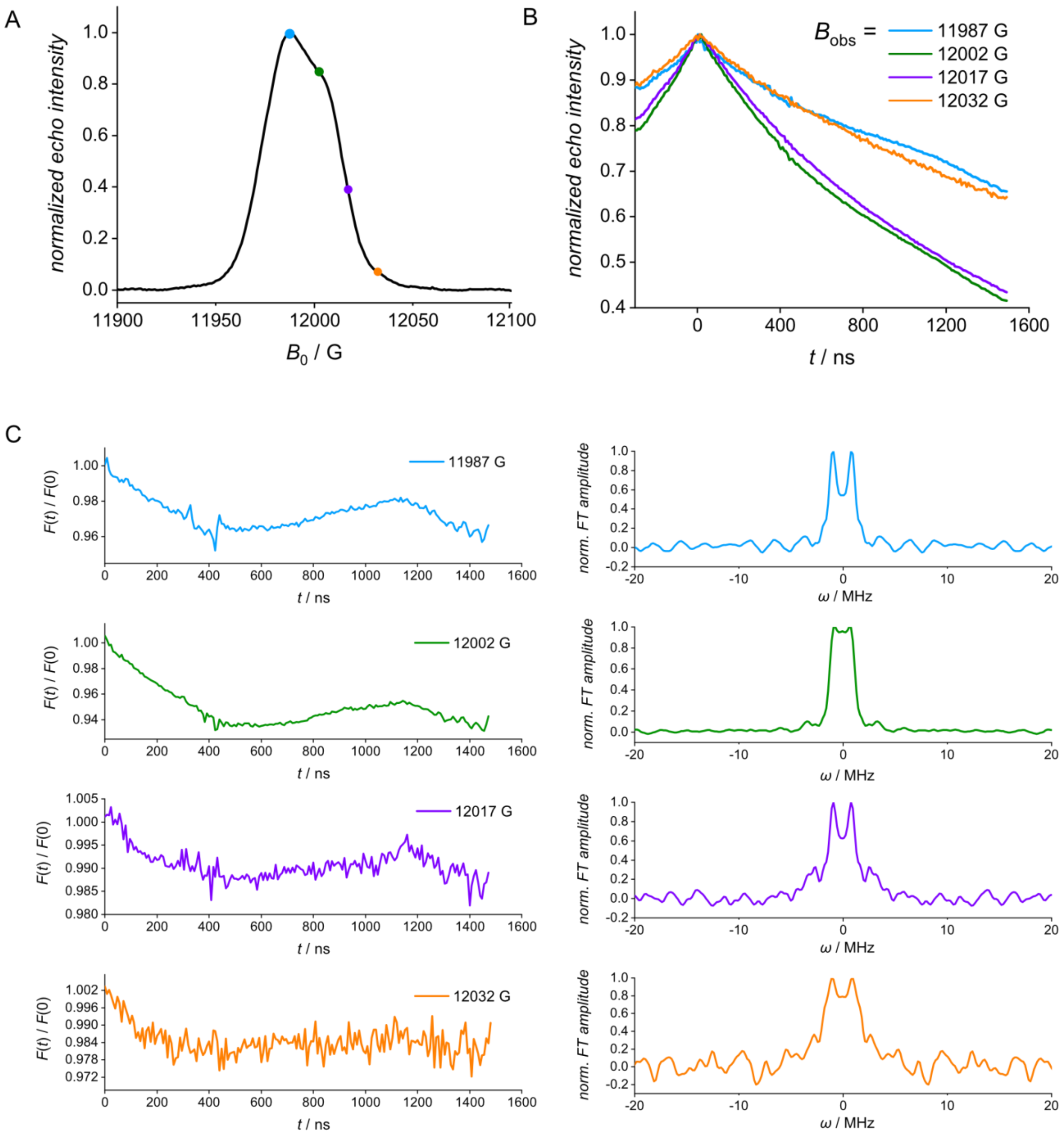

Figure S12. A) EDFS spectrum of the 1[FMN$^{\bullet}$-Met]-2[FMN$^{\bullet-}$] construct. The observer positions are shown as colored dots. The pump pulse was applied at $B_{pump} = B_{obs} - 15$ G. B) Raw PELDOR time traces recorded at the indicated observer positions. C) Background-corrected time traces and corresponding frequency spectra. A second order polynomial was used for the background correction.

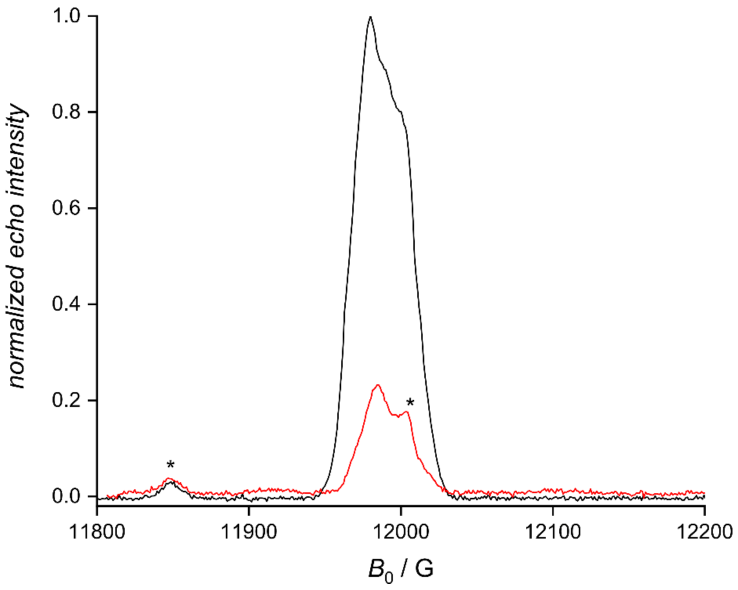

Figure S13. EDFS spectra of 1[FMN•-Met]-2[FMNH•]* *in vitro* (black) after 15 min blue-light irradiation and in cells (red) after 50 min blue-light irradiation at 4 °C, respectively. The spectra were recorded with identical experimental parameters (π-pulse length = 32 ns, τ = 200 ns, power attenuation = 1 dB, SRT = 3.5 ms, 30 spp, video gain = 21 dB). The protein concentration of the *in vitro* sample was determined by absorption spectroscopy to be 165 µM. Resonator background is labeled by asterisks.

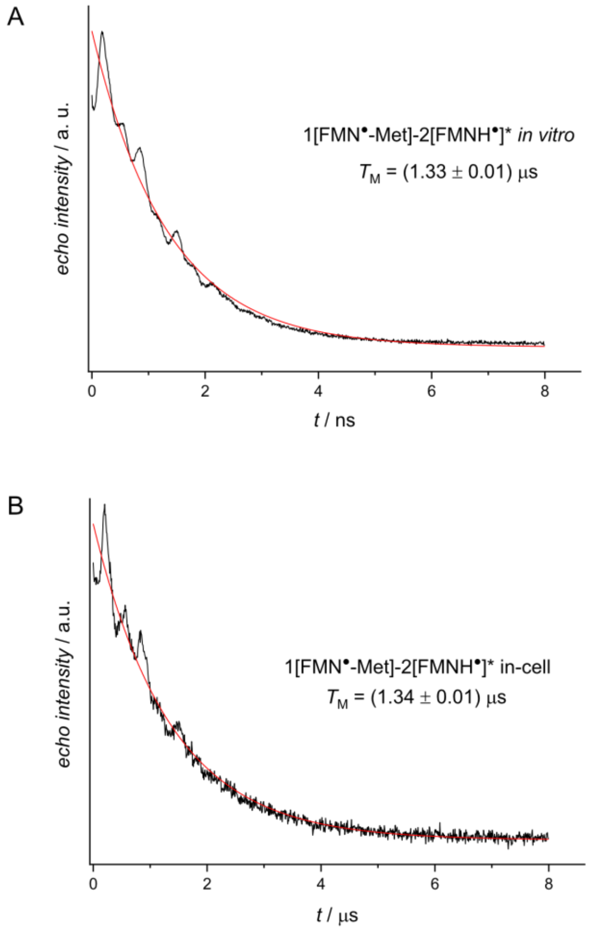

Figure S14. Experimental echo decay traces (black) and corresponding monoexponential fits (red) of the W291F mutant protein *in vitro* (A) and in-cell (B). The time traces were recorded at the maxima of the EDFS spectra shown in Figure S13.

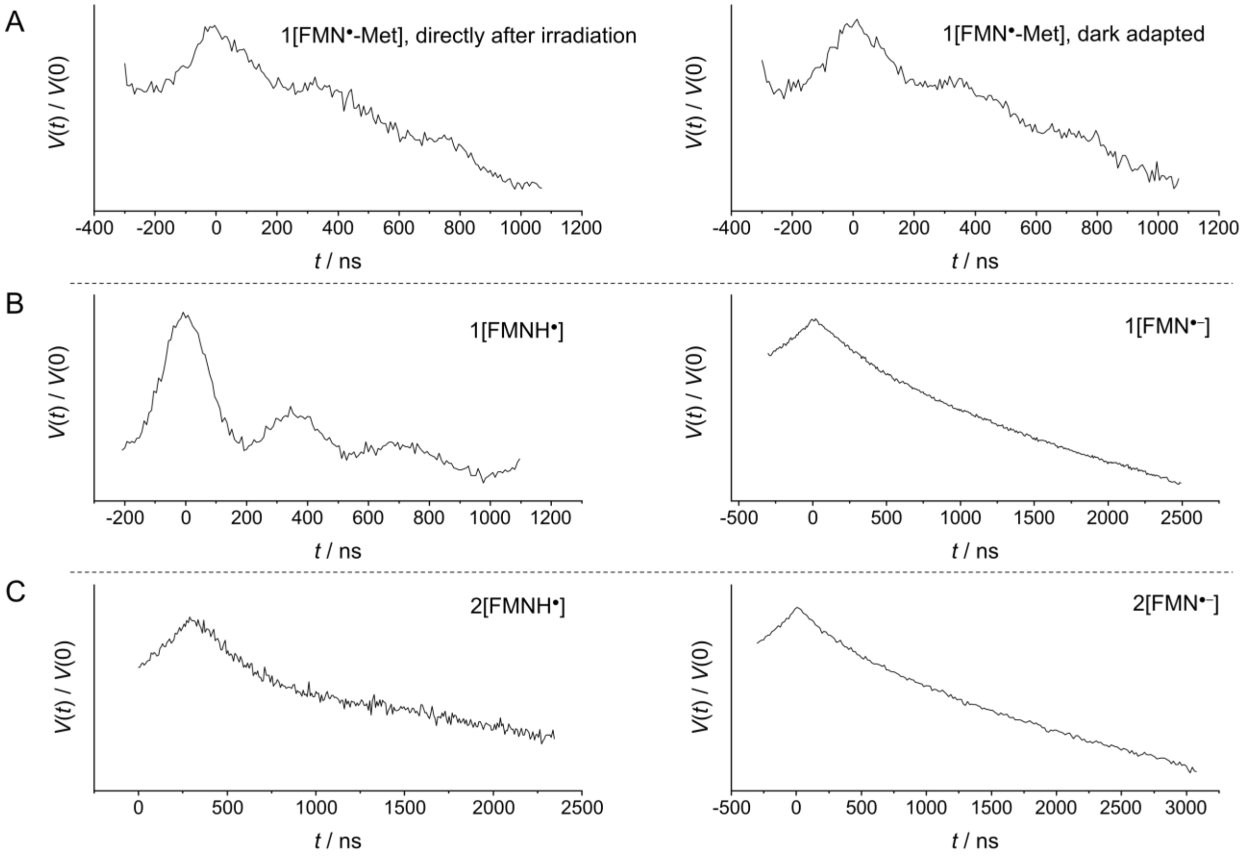

Figure S15. Real components of the raw PELDOR time traces measured on single LOV domains. A) 1[FMN•-Met], B) 1[FMNH•] and 1[FMN•–], C) 2[FMNH•] and 2[FMN•–].

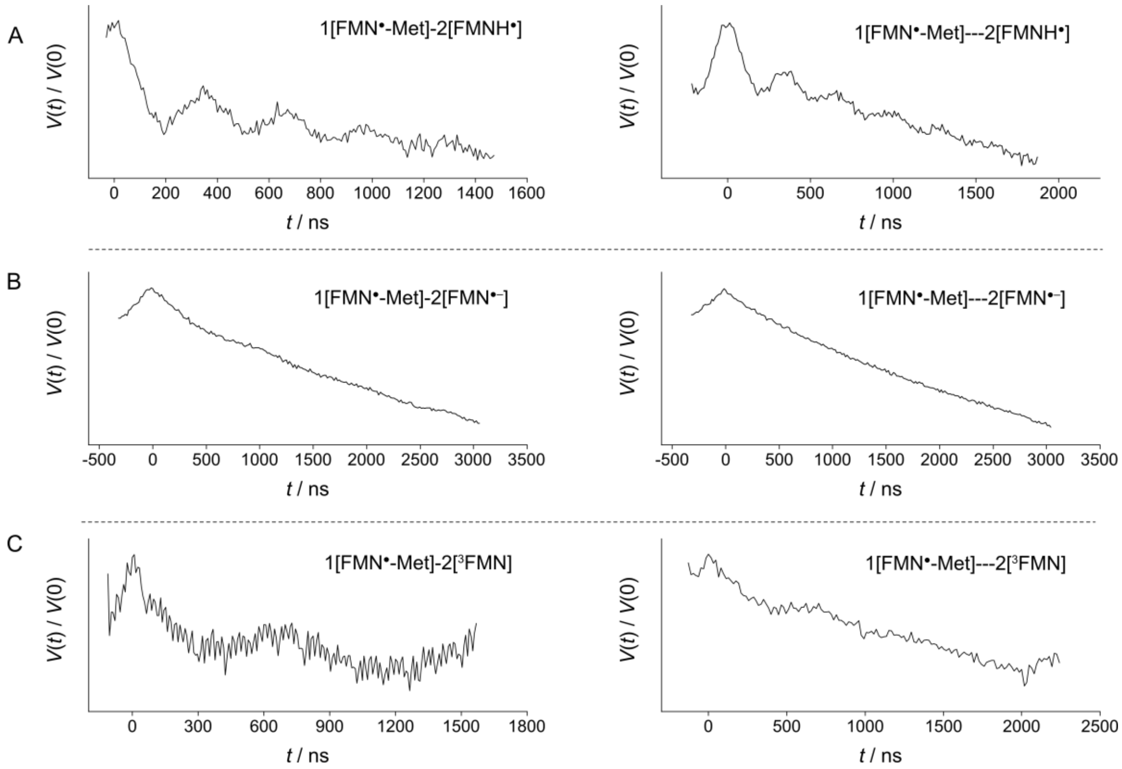

Figure S16. Real components of the raw PELDOR time traces measure on LOV linker constructs. A) PELDOR time traces of the proteins containing two neutral FMN radicals, B) PELDOR time traces of the proteins containing FMN anionic radical in the LOV2 domain, C) ReLaserIMD time traces.

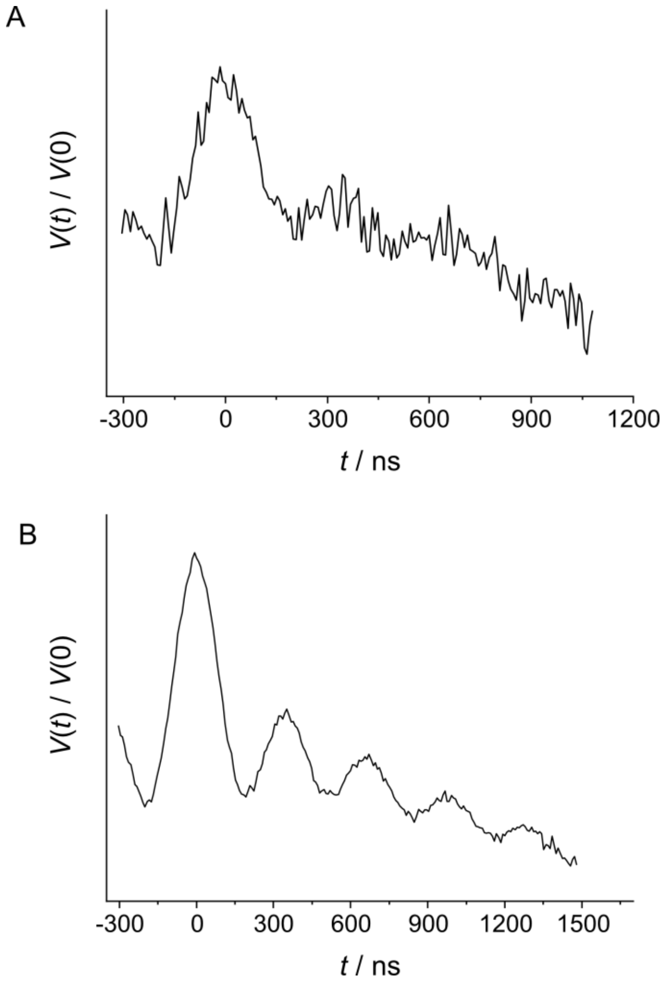

Figure S17. Real components of the raw PELDOR time traces of the 1[FMN$^{\bullet}$-Met]-2[FMNH$^{\bullet}$]* construct in cells (A) and *in vitro* (B).

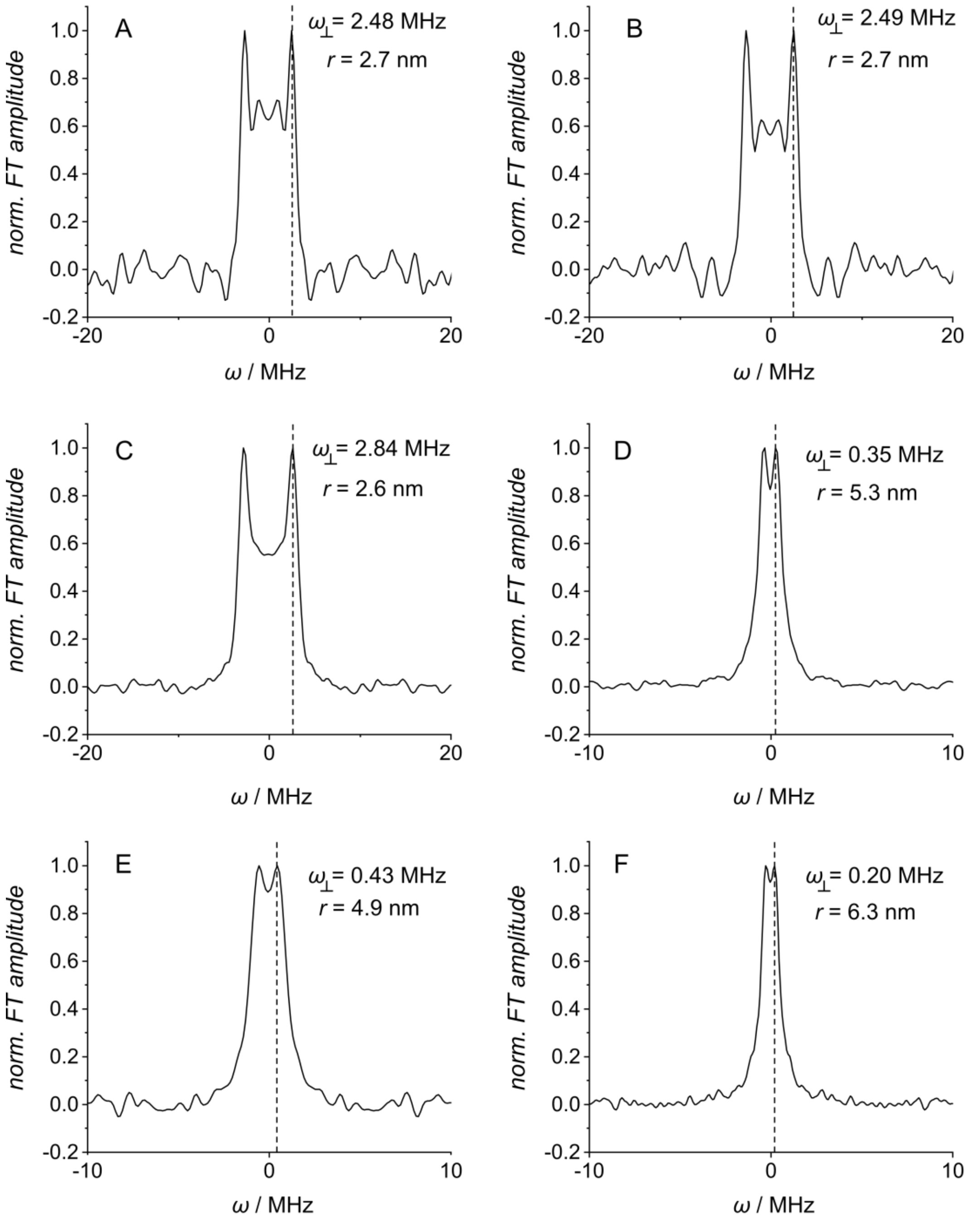

Figure S18. Frequency spectra obtained by Fourier transformation of the PELDOR time traces from LOV single domains (Figure 2 in the main manuscript). A) 1[FMN$^\bullet$-Met] (directly after irradiation), B) 1[FMN$^\bullet$-Met] (dark adapted), C) 1[FMNH$^\bullet$], D) 1[FMN$^{\bullet-}$], E) 2[FMNH$^\bullet$], F) 2[FMN$^\bullet$]. The perpendicular components of the dipolar frequency and the distances, calculated by using the point dipole approximation are denoted.

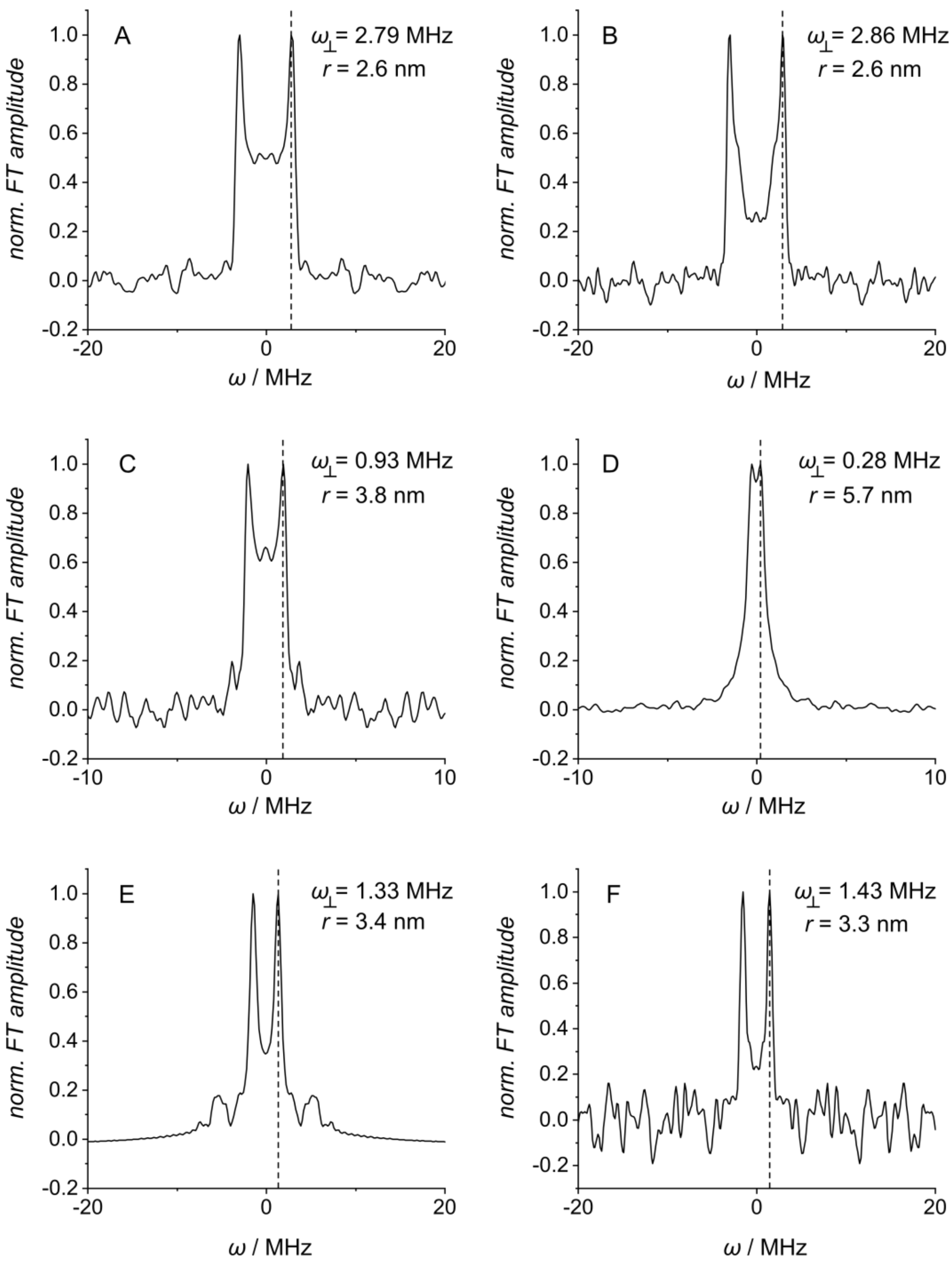

Figure S19. Frequency spectra obtained by Fourier transformation of the dipolar time traces from LOV linker constructs (Figure 3 in the main manuscript). A) 1[FMN$^\bullet$-Met]-2[FMNH$^\bullet$], B) 1[FMN$^\bullet$-Met]---2[FMNH$^\bullet$], C) 1[FMN$^\bullet$-Met]-2[FMN$^{\bullet-}$], D) 1[FMN$^\bullet$-Met]---2[FMN$^{\bullet-}$], E) 1[FMN$^\bullet$-Met]-2[^{3}FMN] (the high frequency noise was removed by applying a high-pass filter), F) 1[FMN$^\bullet$-Met]---2[^{3}FMN]. The perpendicular components of the dipolar frequency and the distances, calculated by using the point dipole approximation are denoted.

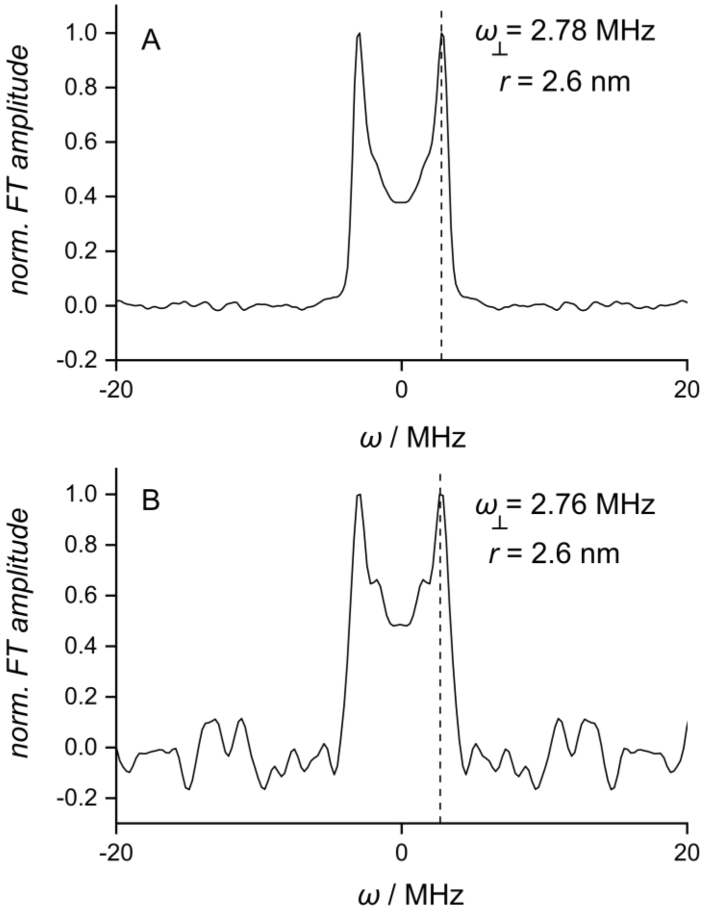

Figure S20. Frequency spectra obtained by Fourier transformation of the dipolar time traces from LOV linker constructs (Figure 3 in the main manuscript). A) 1[FMN•-Met]-2[FMNH•]* (*in vitro*), B) 1[FMN•-Met]-2[FMNH•]* (in cells). The perpendicular components of the dipolar frequency and the distances, calculated by using the point dipole approximation are denoted.

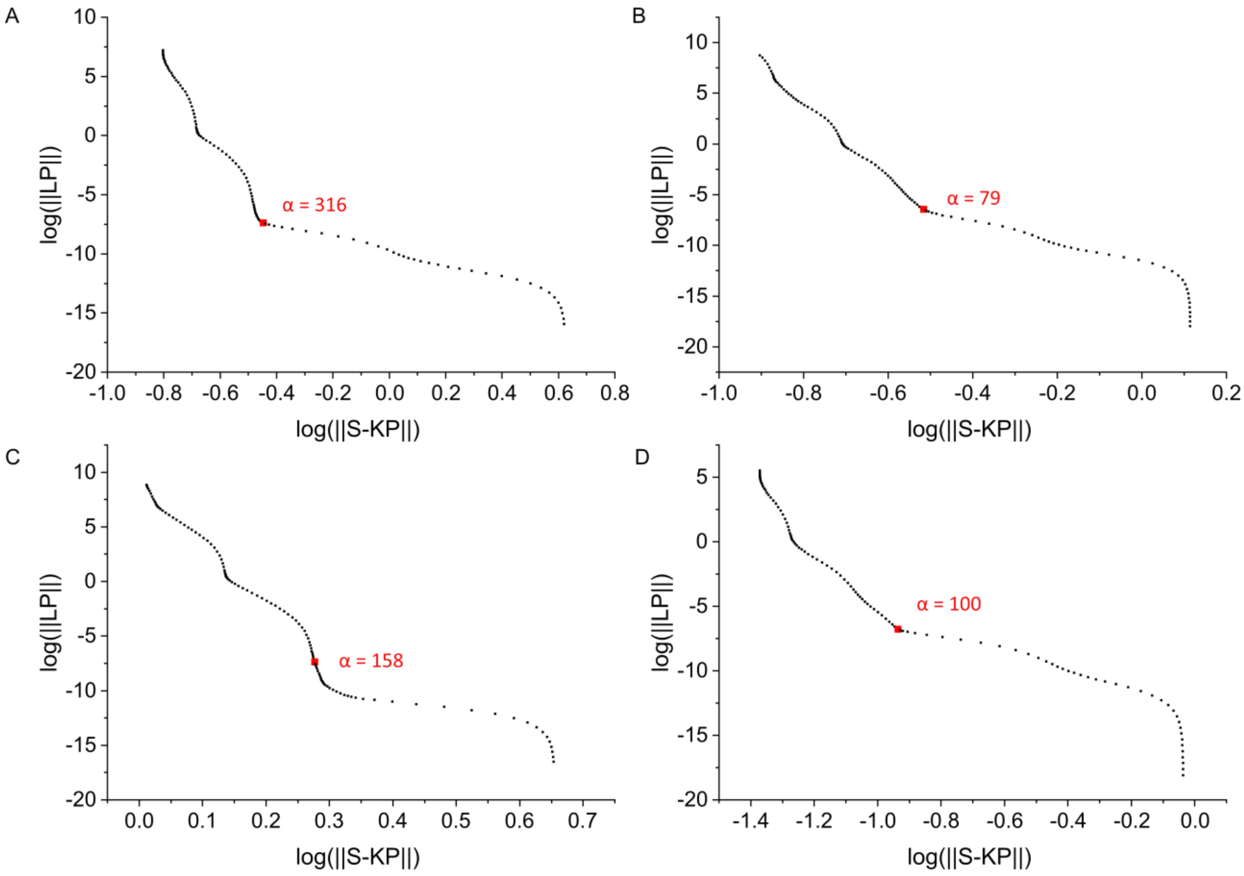

Figure S21. Obtained L-curves for the analysis of the time traces with less pronounced modulations. The depicted regularization parameters in A), B) and D) where chosen by the left corner criterion. The regularization parameter in C) was chosen manually in order to avoid overbroadening of the distance distribution. A) 1[FMN$^{\bullet-}$] (Figure 2D in the main manuscript), B) 2[FMN$^{\bullet-}$] (Fig. 2F in the main manuscript), C) 1[FMNH$^{\bullet}$] (Fig. 2E in the main manuscript), D) 1[FMN$^{\bullet}$-Met]---2[FMN$^{\bullet-}$] (Fig. 3B, bottom part in the main manuscript).

Supporting Table 1. Primers and their respective nucleotide sequences used in this work.

Primer	Nucleotide sequence
LOV1-C57→A (fwd)	5'-GTTCTGGGCCACAATGCTCGTTTTCTGC-3'
LOV1-C57→A (rev)	5'-CAAGACCCGGTGTTACGAGCAAAAGACG-3'
LOV1-C57→M (fwd)	5'-GTTCTGGGCCACAATATGCGTTTTCTGC-3'
LOV1-C57→M (rev)	5'-CAAGACCCGGTGTTATACGCAAAAGACG-3'
LOV2-W291→F(fwd)	5'-GCGGGCAAAGCGTTCTTCAACATGTTTACCCTGGC-3'
LOV2-W291→F(rev)	5'-GCAAGGGTAAACATGTTGAAGAACGCTTTGCCCGC-3'
Sequencing primer	5'-TAATACGACTCACTATAGGGGAATTGTG-3'

Supporting Table 2. Modulation depths and signal-to-noise ratios of the dipolar time traces presented in the main manuscript. The SNR was calculated by dividing the modulation depth by the residual of the imaginary part after phase correction.[3]

Sample	modulation depth / %	Signal/noise ratio	Figure in the main manuscript
**1[FMN$^\bullet$-Met]**	1.6 and 2.5	3.3 and 4.2	2A and 2B
**1[FMNH$^\bullet$]**	8.9	7.1	2C
**1[FMN$^{\bullet-}$]**	5.3	19.7	2D
**2[FMNH$^\bullet$]**	4.3	6.5	2E
**2[FMN$^{\bullet-}$]**	4.5	7.4	2F
**1[FMN$^\bullet$-Met]-2[FMNH$^\bullet$]**	17.8	11.1	3A (top)
**1[FMN$^\bullet$-Met]---2[FMNH$^\bullet$]**	11.4	8.3	3A (bottom)
**1[FMN$^\bullet$-Met]-2[FMN$^{\bullet-}$]**	5.4	13.4	3B (top)
**1[FMN$^\bullet$-Met]---2[FMN$^{\bullet-}$]**	5.7	8.0	3B (bottom)
**1[FMN$^\bullet$-Met]-2[^{3}FMN]**	0.5	0.5	3C (top)
**1[FMN$^\bullet$-Met]---2[^{3}FMN]**	0.4	0.2	3C (bottom)
**1[FMN$^\bullet$-Met]-2[FMNH$^\bullet$]* *in vitro***	17.4	32.2	4B
**1[FMN$^\bullet$-Met]-2[FMNH$^\bullet$]* in cell**	2.4	3.8	4A

References

(1) Fedorov, R.; Schlichting, I.; Hartmann, E.; Domratcheva, T.; Fuhrmann, M.; Hegemann, P. Crystal structures and molecular mechanism of a light induced signaling switch: the phot-LOV1 domain from *Chlamydomonas reinhardtii*. *Biophysical Journal* **2003**, *84*, 2474-2482.
(2) Tait, C. E.; Krzyaniak, M. D.; Stoll, S. Computational tools for the simulation and analysis of spin-polarized EPR spectra. *Journal of Magnetic Resonance* **2023**, *349*, 107410.
(3) Brandon, S.; Beth, A. H.; Hustedt, E. J. The global analysis of DEER data. *Journal of Magnetic Resonance* **2012**, *218*, 93-104. Schiemann, O.; Heubach, C. A.; Abdullin, D.; Ackermann, K.; Azarkh, M.; Bagryanskaya, E. G.; Drescher, M.; Endeward, B.; Freed, J. H.; Galazzo, L.; et al. Benchmark test and guidelines for DEER/PELDOR experiments on nitroxide-labeled biomolecules. *Journal of the American Chemical Society* **2021**, *143*, 17875-17890.
